# Behavioral interventions motivate action to address climate change

**DOI:** 10.1073/pnas.2426768122

**Published:** 2025-05-13

**Authors:** Alyssa H. Sinclair, Danielle Cosme, Kirsten Lydic, Diego A. Reinero, José Carreras-Tartak, Michael E. Mann, Emily B. Falk

**Affiliations:** ^a^Penn Center for Science, Sustainability, and the Media, University of Pennsylvania, Philadelphia, PA 19104; ^b^Annenberg Public Policy Center, University of Pennsylvania, Philadelphia, PA 19104; ^c^Annenberg School for Communication, University of Pennsylvania, Philadelphia, PA 19104; ^d^Department of Psychology, University of Pennsylvania, Philadelphia, PA 19104; ^e^Department of Earth and Environmental Sciences, University of Pennsylvania, Philadelphia, PA 19104; ^f^Wharton Marketing Department, University of Pennsylvania, Philadelphia, PA 19104; ^g^Wharton Operations, Information and Decisions Department, University of Pennsylvania, Philadelphia, PA 19104

**Keywords:** climate change, psychology, behavior change, pro-environmental behavior, information sharing

## Abstract

Mitigating climate change requires urgent action at individual, collective, and institutional levels. However, individuals may fail to act because they perceive climate change as a threat that is distant or not personally relevant, or believe their actions are not impactful. To address these psychological barriers, we conducted a large-scale “intervention tournament.” In a sample of 7,624 participants, we systematically tested 17 interventions that targeted psychological mechanisms described by three key themes: Relevance, Future Thinking, and Response Efficacy. Interventions that emphasized social relevance were the most effective for motivating people to share news articles and petitions about climate change. Interventions that targeted future thinking were the most effective for broadly motivating individual actions (e.g., driving less, eating vegetarian meals) and collective actions (e.g., donating, volunteering) to address climate change. Interventions that emphasized the environmental impact of these actions reliably increased the perceived impact of pro-environmental actions, but did not consistently motivate action. Notably, interventions that targeted two or more mechanisms—such as imagining a future scenario that involved oneself or close others—were most effective. Importantly, our leading interventions were substantially more effective than prevalent existing strategies (e.g., carbon footprint information). Our findings are relevant to theories of behavior change, motivation, and information sharing, with potential applications across domains. Insights from our tournament could be applied to develop scalable online interventions and mass communication campaigns to address climate change.

## Significance

Climate change poses an urgent threat to humans and ecosystems globally; human behavior is both cause and solution. Although a majority of people believe that climate change is occurring, many fail to take action. We conducted an intervention tournament, systematically testing 17 psychological strategies to motivate people to share information about climate change and take action in daily life. Our tournament offers insights into which strategies are most effective and why, identifying key mechanisms of action. Our findings are relevant to psychological theories of behavior change, motivation, decision making, learning, and information sharing. Crucially, our leading interventions could be readily scaled to develop accessible and engaging tools for climate change communication, of relevance to communicators, policymakers, and environmental scientists.

## Behavioral Interventions Motivate Action to Address Climate Change

Climate change poses an urgent, global threat to the health and well-being of humans, other species, and ecosystems. This crisis can be addressed by changing human behavior at individual, collective, and institutional levels (1). Approximately 72% of people in the United States (2) and 85% of people worldwide (3, 4) believe that climate change is occurring, though beliefs about the causes of climate change (anthropogenic vs. natural) also vary within this group. Despite this widespread acknowledgement of climate change, multiple psychological and structural barriers impede climate action (5–8). For instance, individuals may struggle to relate climate change to themselves and people they know, perceive climate change as an abstract future threat, or believe that their actions are not efficacious (5–8). To address these barriers, we developed a set of interventions that targeted interrelated psychological mechanisms under three key themes: Relevance, Future Thinking, and Response Efficacy. We conducted an *intervention tournament* to systematically test these intervention strategies, aiming to increase intentions to engage in pro-environmental behaviors, the perceived impact of pro-environmental behaviors, and intentions to share information about climate change.

## Psychological Factors Influencing Climate Change Beliefs and Behaviors

### Perceived Relevance.

Research suggests that people’s perceptions of self- and social-relevance determine their actions (9–12). People may fail to take action because climate change may not seem relevant to themselves or people they know (13–15). For example, approximately 40% of Americans report little-to-no impact of climate change in their communities, and do not expect to see much impact in the next 30 y (16, 17). Inaccurate perceptions of social norms can also create the illusion threat climate change is not important or relevant to most other people. Such pluralistic ignorance regarding climate change has been shown in the United States (18) and worldwide (19, 20). These gaps can lead to a “climate of silence,” which exacerbates misperceptions of social norms (15). These perceptions have downstream consequences; individuals who view climate change as a socially distant problem report lower concern and policy support (21).

Recognizing the self- and social-relevance of climate change could motivate people to share information and take action. Converging correlational and causal evidence indicates that when people perceive information as relevant to themselves or close others, they are more likely to value that information and share it with others (10, 11, 22–26). Sharing information about climate change could help address pluralistic ignorance gaps by changing perceived social norms. Social norms have been shown to be a powerful motivator for behavior change across many domains (27, 28), including for climate action (14, 29–34), health (35, 36), and reducing group conflict (37, 38). Other interventions have demonstrated that reducing the perceived social distance of climate change (i.e., learning how climate change will impact people like oneself) can increase concern and policy support (21, 39, 40). Interventions that highlight the self- and social-relevance of climate change or provide information about social norms could therefore address these barriers.

### Future Thinking.

A second body of work highlights the promise of future thinking interventions for motivating action. Across domains, people tend to demonstrate a present bias, overvaluing immediate rewards relative to long-term consequences (41, 42). Such temporal discounting may lead individuals to devalue the future threats of climate change. Addressing climate change requires immediate action for long-term gain, much like investing money for retirement instead of spending it (43–45). However, the present bias can be harnessed in service of long-term goals when immediate rewards increase motivation and perseverance (46–48). Imagination exercises can also shift the balance between short-term and long-term priorities, encouraging future-oriented decision making (49, 50). Such imagination exercises have been used to change risk perception and action intentions (51, 52), motivate pro-environmental behaviors (53), and increase prosocial behavior (54, 55). Relatedly, imagining and planning the steps required to achieve a future goal motivates action (56).

Thinking about the future could also motivate action by reducing the psychological distance of climate change. Prior evidence suggests that psychological distance predicts beliefs, concern, action intentions, and policy support (21, 39, 57, 58). However, other studies have shown inconsistent effects (59–61). These mixed findings suggest that targeting multiple aspects of psychological distance, such as temporal and social distance, may be more effective. Supporting this idea, prior studies have shown that thinking about one’s intergenerational legacy reduces psychological distance and motivates climate action (62, 63). Similarly, emphasizing one’s moral responsibility to care for future generations is associated with pro-environmental support (64, 65). Taken together, these studies suggest that imagining future actions and outcomes—for oneself and for future generations—may effectively motivate climate action.

### Response Efficacy.

A third body of research suggests that interventions should aim to increase response efficacy, highlighting the positive impact of actions. Beliefs about one’s ability to enact specific behaviors (self-efficacy) and beliefs about the downstream impact of those actions (response efficacy) shape intentions (66, 67). Even individuals who are concerned about climate change may fail to take action because they believe that their actions do not matter. Climate change is a complex systems problem (68, 69) that must be addressed with collective action (70). It is difficult to understand or observe the impact of our actions, which may make individuals feel that their contributions are insignificant. Feeling capable of enacting change is associated with action intentions, across domains (67, 71, 72) and for climate change specifically (73–76). Illustrating the cumulative, downstream impact of changing everyday behavior may help people realize that their seemingly small actions do matter, and providing skills coaching can make people feel more confident in their ability to change (77, 78). A related barrier is that individuals may be unsure which actions matter most, reducing their response efficacy. Beliefs about the impact of pro-environmental behaviors are poorly aligned with recommendations from experts (79). Individuals favor low-impact actions like recycling over high-impact actions like reducing air travel, and misestimate the energy savings associated with various actions (80, 81). Correcting misconceptions about impact could thus direct effective action by increasing the response efficacy of high-impact behaviors.

Taken together, these diverse bodies of evidence demonstrate that multiple psychological factors—including relevance, future thinking, and response efficacy—can impede or motivate action to address climate change. Importantly, these factors can be interrelated. For example, envisioning future outcomes for oneself and close others could increase perceived relevance while also facilitating future-oriented thinking and illustrating the downstream impact of actions. Interventions that target multiple psychological factors may be particularly effective for motivating behavior change.

## Identifying and Comparing Effective Interventions

To address the climate crisis, we urgently need evidence-based, scalable strategies for motivating action. Online interventions could reach broad audiences to motivate individuals to share information, talk to others about climate change, make lifestyle changes, donate, vote, or sign petitions. In addition to developing effective interventions, it is crucial to understand which interventions are ineffective or harmful. For instance, interventions that quantify individuals’ carbon footprints are widely promoted by major environmental agencies like the U.S. Environmental Protection Agency (82) and the World Wildlife Fund (83) even though this approach was developed by British Petroleum (84) and there is little empirical evidence of effectiveness (85, 86). Indeed, an argument can be made that overly focusing on individual carbon footprints can reduce the perceived urgency of systemic efforts (e.g., policy incentives for decarbonization) (87). Positive, null, and negative intervention effects are all valuable and informative for changing the landscape of climate communication.

Given the broad spectrum of psychological factors that may motivate behavior change (6), it is essential to systematically test and compare psychological interventions against common benchmarks. Evidence from prior studies pertaining to climate change is mixed and inconclusive, potentially because of differences in task design, outcome measures, construct definitions, study population, and time of year (88). An intervention tournament approach (89), in which ideas from multiple sources are tested simultaneously on the same outcome measures, is ideal for overcoming these limitations. The tournament approach enables researchers to assess the relative strength of different intervention strategies against a standardized set of outcomes.

A recent global study used an intervention tournament to test and compare 11 behavioral interventions for climate change (4). This work laid an important foundation for testing light-touch behavioral interventions, focusing on four key outcomes: beliefs, policy support, information sharing, and action. Results indicated that intervention effects differed considerably across audiences and target behaviors, and effect sizes were small. Notably, some of the most effective interventions for one outcome (e.g., information sharing) had robust backfire effects on other outcomes (e.g., climate action). None of the interventions tested in this tournament increased climate action, and several of the interventions decreased action. Overall, this recent tournament identified several promising intervention strategies and investigated cross-cultural differences. These recent findings also highlight a key gap—future studies must test additional intervention strategies to motivate climate action and identify ways to motivate information sharing without producing backfire effects.

## Present Study

We conducted a large-scale intervention tournament to systematically compare the effectiveness of interventions that are grounded in psychological and neural models of belief and behavior change (67, 90–92). Importantly, our tournament differs from and builds on evidence from a recent tournament with similar aims (4) in several ways: We used a theory-driven approach to systematically test interventions that target key psychological mechanisms and aim to engage brain systems implicated in choice and behavior change, tested a distinct set of intervention strategies, collected additional measures to elucidate mechanisms, and identified interventions that motivated information sharing and climate action without causing backfire effects.

Our interventions integrated and compared theoretical predictions from psychology, neuroscience, and communication science. We aimed to compare effect sizes across these theoretically grounded interventions, using a data-driven approach to identify leading strategies. Although we did not forecast an overall “winner” of the tournament, we preregistered methods and predictions for most individual interventions. Notably, these preregistrations include some additional intervention-specific analyses that are beyond the scope of this report but will be included in separate reports (https://osf.io/x9c6j/registrations).

We generally expected that all interventions would increase intentions to share information about climate change, intentions to engage in pro-environmental behaviors, and the perceived impact of these behaviors, relative to the no-intervention control group. Furthermore, we expected that the three psychological mechanisms targeted by our interventions—Relevance, Future Thinking, and Response Efficacy—would preferentially correspond to our three primary outcomes. We predicted the following: 1) Interventions that targeted Relevance would increase intentions to share information about climate change; 2) Interventions that targeted Future Thinking would increase intentions to engage in pro-environmental behaviors, and 3) Interventions that targeted Response Efficacy would increase the perceived impact of pro-environmental behaviors. We also expected that all interventions had the potential to be effective for any or all of our primary outcome measures, particularly interventions that targeted multiple psychological mechanisms. We did not make specific predictions about relative effect sizes across interventions.

### Tournament Design.

**Fig. 1.**
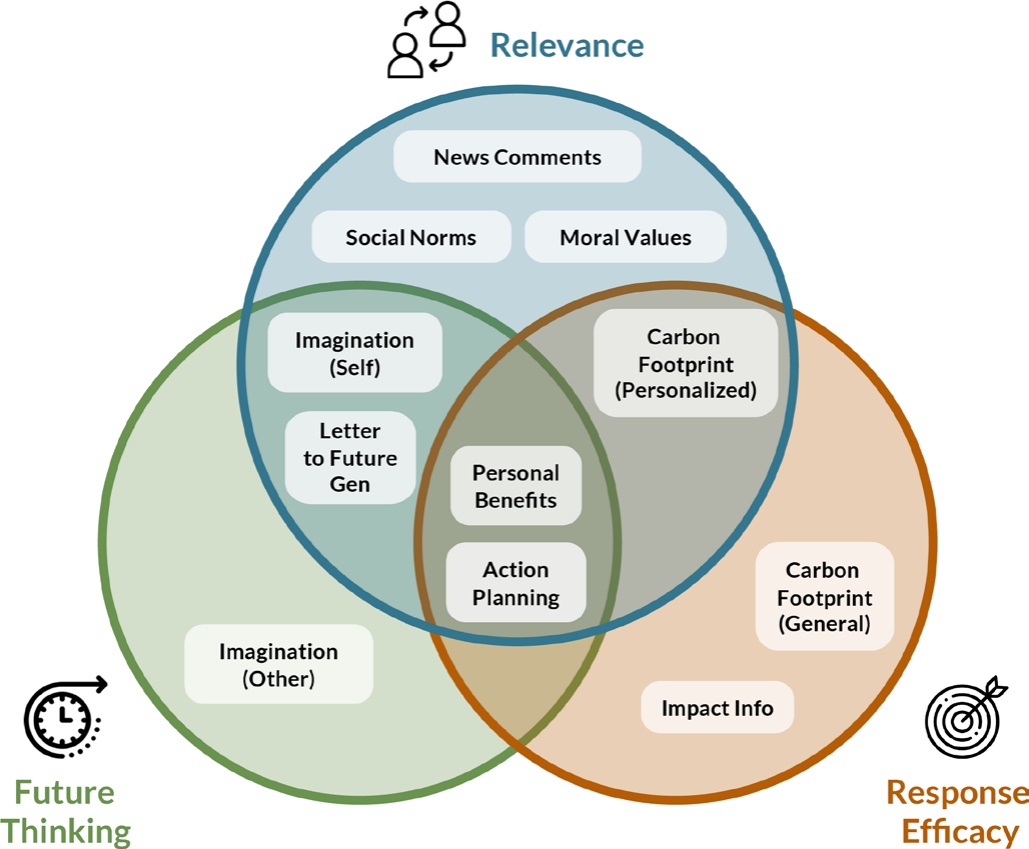
Overview of interventions tested in the tournament, organized into three key themes: Relevance (*Top*), Future Thinking (*Left*), and Response Efficacy (*Right*). Some interventions, indicated in overlapping portions of the theme circles, leveraged multiple psychological mechanisms.

**Fig. 2.**
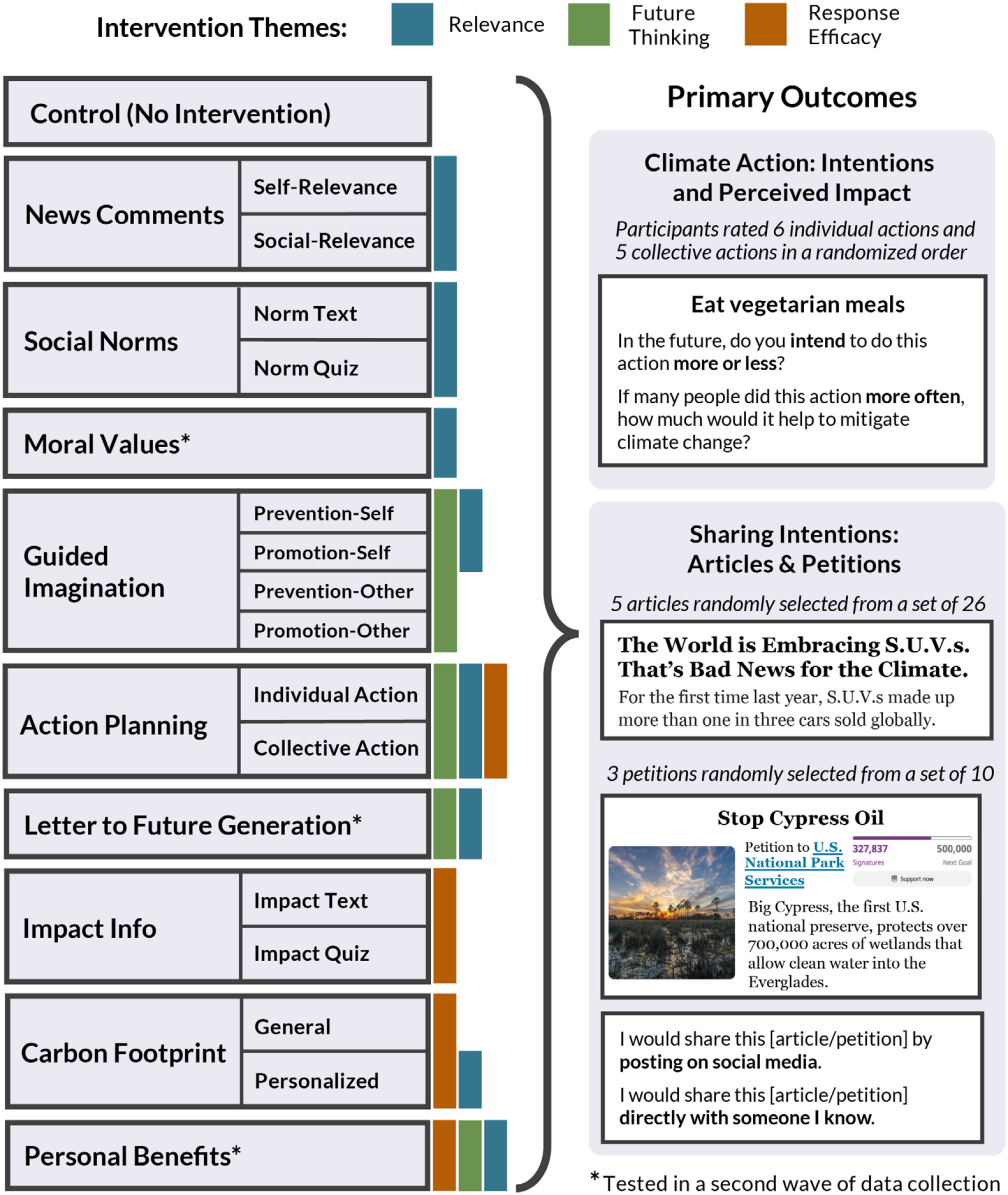
Overview of the intervention tournament. The *Left* panel lists all interventions tested; for some intervention strategies, we tested multiple variations. Where applicable, these subgroups are labeled in smaller boxes to the right of each parent intervention label. Interventions targeted different psychological mechanisms, indicated here with three color-coded themes: Relevance, Future Thinking, and Response Efficacy. Some interventions targeted multiple mechanisms (Fig. 1), marked with multiple colored bars to the right of each intervention box. For multitheme interventions, the leftmost box indicates the primary theme. The Control group did not complete any intervention task, and proceeded directly to completing the outcome measures after providing consent. Participants were randomly assigned to a group in a between-subjects design. The *Right* panel illustrates the primary outcome measures: ratings of future intentions and perceived impact regarding pro-environmental individual and collective actions related to climate change, and intentions to share news headlines and petitions about climate change. All participants completed the same set of outcome measures. In addition to the primary outcomes illustrated here, participants also completed a battery of secondary outcome measures, described in detail in *SI Appendix*. * denotes intervention conditions that were tested in a second wave of data collection; all interventions were compared with the same control group for consistency.

We recruited 7,624 U.S. adults and randomly assigned them to one of 17 intervention conditions or a no-intervention control group in a between-subjects design. Our interventions tested different tactics intended to engage one or more of the key psychological mechanisms, aligning with the three overarching themes (Figs. 1 and 2). To determine the most effective implementation of each intervention strategy, in some cases, we tested multiple variations within each “parent” intervention. Although some interventions targeted multiple mechanisms, for simplicity, below we group interventions according to the primary theme for each intervention. Additional information is provided in *Materials and Methods* and *SI Appendix*.

Interventions under the Relevance theme targeted perceived self- and social-relevance as a psychological mechanism. These interventions aimed to relate climate change to oneself and close others. In the News Comments interventions, participants wrote brief comments regarding news headlines about climate change, describing why the headlines mattered to them (Self-Relevance condition, n = 396), or mattered to people they knew (Social-Relevance condition, n = 392). In the Social Norm Information interventions, participants viewed statistics about normative attitudes (e.g., belief in climate change, policy support, willingness to make lifestyle changes), either as an interactive quiz with feedback (Norm Quiz condition, n = 426), or as descriptive statements (Norm Text condition, N = 428). In the Moral Values intervention (N = 420), participants identified their most important moral value from a list, then completed a writing exercise and read a message that related their chosen moral value to climate change.

Interventions under the Future Thinking theme targeted future-oriented cognition as a psychological mechanism, such as by illustrating the potential long-term consequences of climate change and pro-environmental behaviors for the self and others. In the Guided Imagination interventions, participants completed a structured imagination and writing exercise. Participants imagined one of four scenarios; we varied whether participants imagined themselves or a fictional character experiencing a negative future that could result from failure to address climate change (Prevention-Self condition, n = 380; Prevention-Other condition, n = 374) or a positive future that could result from climate action (Promotion-Self condition, n = 373; Promotion-Other condition, n = 374). In the Action Planning interventions, participants chose a personal climate action goal and developed a detailed plan to achieve it, imagining the steps involved, potential obstacles, and outcomes. Participants selected a target action from a list of individual actions (Individual Action Planning condition, n = 393), such as flying less or driving less, or a list of collective actions (Collective Action Planning condition, n = 382), such as donating to or volunteering for climate-related organizations. In the Letter to Future Generation intervention (N = 391), participants wrote a letter to a socially close child as if the recipient would read this letter in the future, as an adult. In the letter, participants described their aspirations and efforts to ensure that the child would inherit a habitable planet.

Interventions under the Response Efficacy theme targeted beliefs about impact as a psychological mechanism, such as by emphasizing the potential benefits of pro-environmental behaviors, for the environment or for oneself. In the Impact Information interventions, participants learned about the environmental impact (estimated reduction of greenhouse gas emissions) of actions that individuals could take to mitigate climate change, either by completing a quiz with feedback (Impact Quiz condition, n = 416) or reading descriptive statements (Impact Text condition, n = 418). In the Carbon Footprintinterventions, participants either received general information about how lifestyle changes can reduce one’s carbon footprint (General Carbon Footprint condition, n = 428), or completed a lifestyle survey and received personalized feedback about how various actions would reduce their carbon footprints (Personalized Carbon Footprint condition, n = 413). In the Personal Benefits intervention (n = 370), participants brainstormed short-term *personal* benefits (e.g., improving health, happiness, relationships, or finances) that could arise from engaging in pro-environmental behaviors over the next six months.

### Outcome Measures.

In evaluating the effects of our interventions, we focused on three primary outcome measures: *Intentions to engage in pro-environmental behaviors*, the *perceived impact* of the same pro-environmental behaviors, and *intentions to share information* about climate change.

We measured behavioral intentions and perceived impact using a Climate Action Task. In this task, participants answered questions about seven individual actions (e.g., eating vegan meals, paying for renewable energy at home) and five collective actions (e.g., volunteering, donating) related to climate change. Importantly, these target behaviors were both feasible for individuals (as identified in a pilot study) and impactful for addressing climate change (in terms of estimated reduction of greenhouse gas emissions) (93). For each action, participants reported their current frequency of engaging in the action and their intentions to engage in the action more or less in the future (1 = *a lot less*, 7 = *a lot more*). Participants also rated the perceived impact of each action (i.e., collective efficacy beliefs), estimating the beneficial environmental impact if many people engaged in a particular action (1 = *no impact*, 7 = *very large impact*).

We measured intentions to share information with measures collected in two different tasks. In separate tasks, participants viewed five news headlines about climate change (sourced from *The New York Times*) and three petitions about climate change (sourced from *change.org*). For each headline or petition, participants used a scale ranging from 0 (*strongly disagree*) to 100 (*strongly agree*) to rate their intentions to share the information broadly on social media and directly with people they knew.

We also included other measures that were intended to investigate psychological mechanisms of action and other intervention effects. These secondary measures included self-efficacy beliefs, emotions related to climate change, psychological distance of climate change, perceived risk of climate change, perceived self- and social-relevance of climate information, and intentions to sign petitions. Detailed results for secondary outcome measures are reported in *SI Appendix*, *Supplementary Results* and visually summarized in *SI Appendix*, Table S9.

### Analysis Approach.

For all analyses, we used Bayesian linear regression models to compare each outcome measure across conditions (17 intervention groups and the Control group). For outcome measures with multiple observations per participant (e.g., all measures from the Climate Action, News Headlines, and Petitions tasks), we used mixed-effects models that accounted for variance within participants and items (*SI Appendix*, *Statistical Analysis*). Notably, the model predicting action intentions also included a covariate to account for current frequency of engaging in each behavior. We compared point estimates (median of posterior distribution) for each intervention condition with the Control condition; we consider an intervention effect significantly different from the Control group if the lower bound of the 95% credible interval is greater than the Control group point estimate. Further information about statistical analysis is provided in the Methods.

## Results

We investigated whether the interventions increased intentions to engage in pro-environmental behaviors, the perceived impact of these behaviors, and intentions to share information about climate change. Results are visually summarized in Table 1. Descriptive statistics for all primary outcomes in the Control group are provided in *SI Appendix* as a reference (*SI Appendix*, Table S2).

**Table 1.** Summary table of results for primary outcome measures

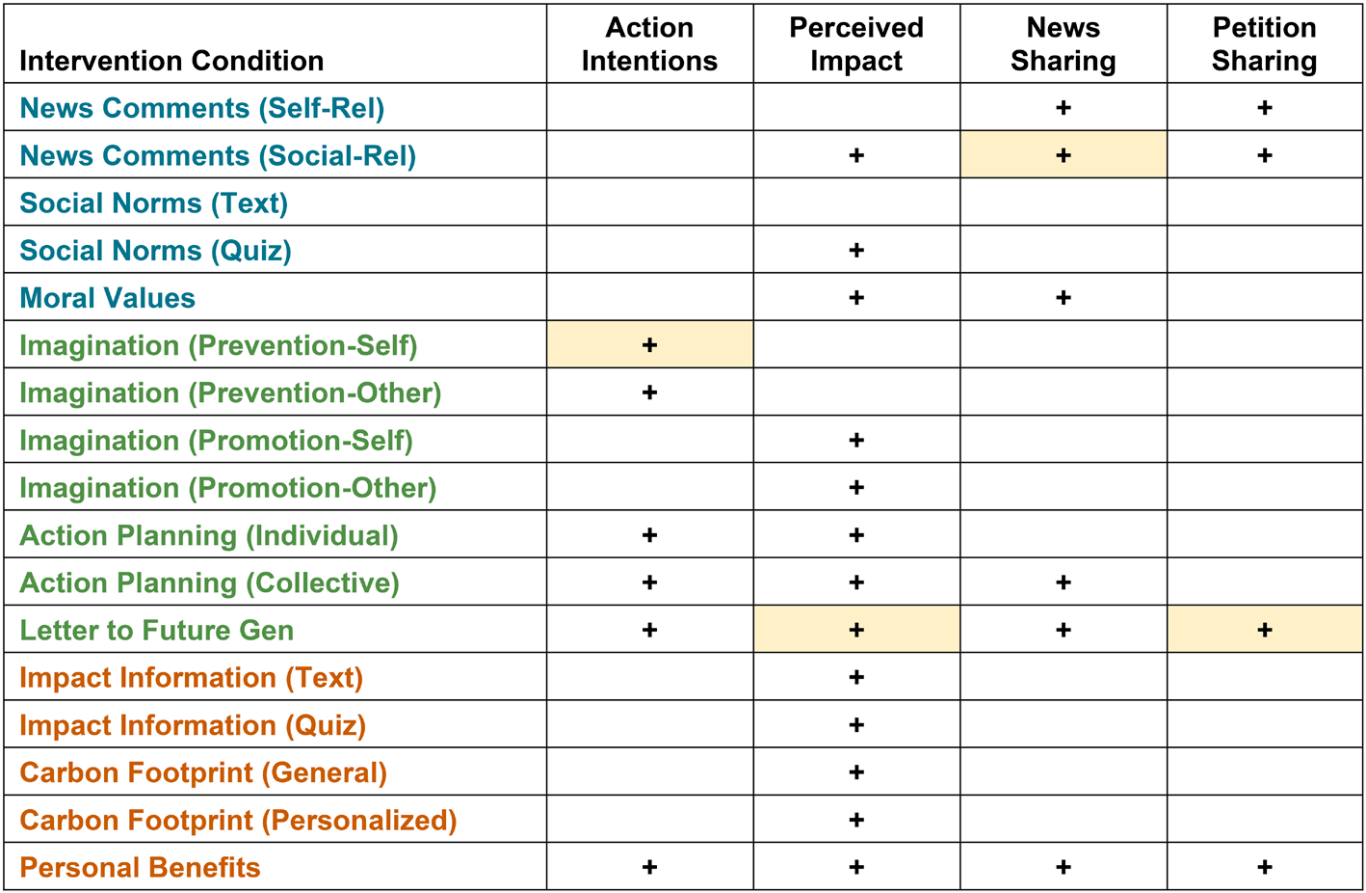

+ indicates a significant intervention effect (greater than Control group). Shaded cells identify the intervention with the strongest effect for each outcome measure.

### Intentions to Engage in Pro-Environmental Behaviors.

**Fig. 3.**
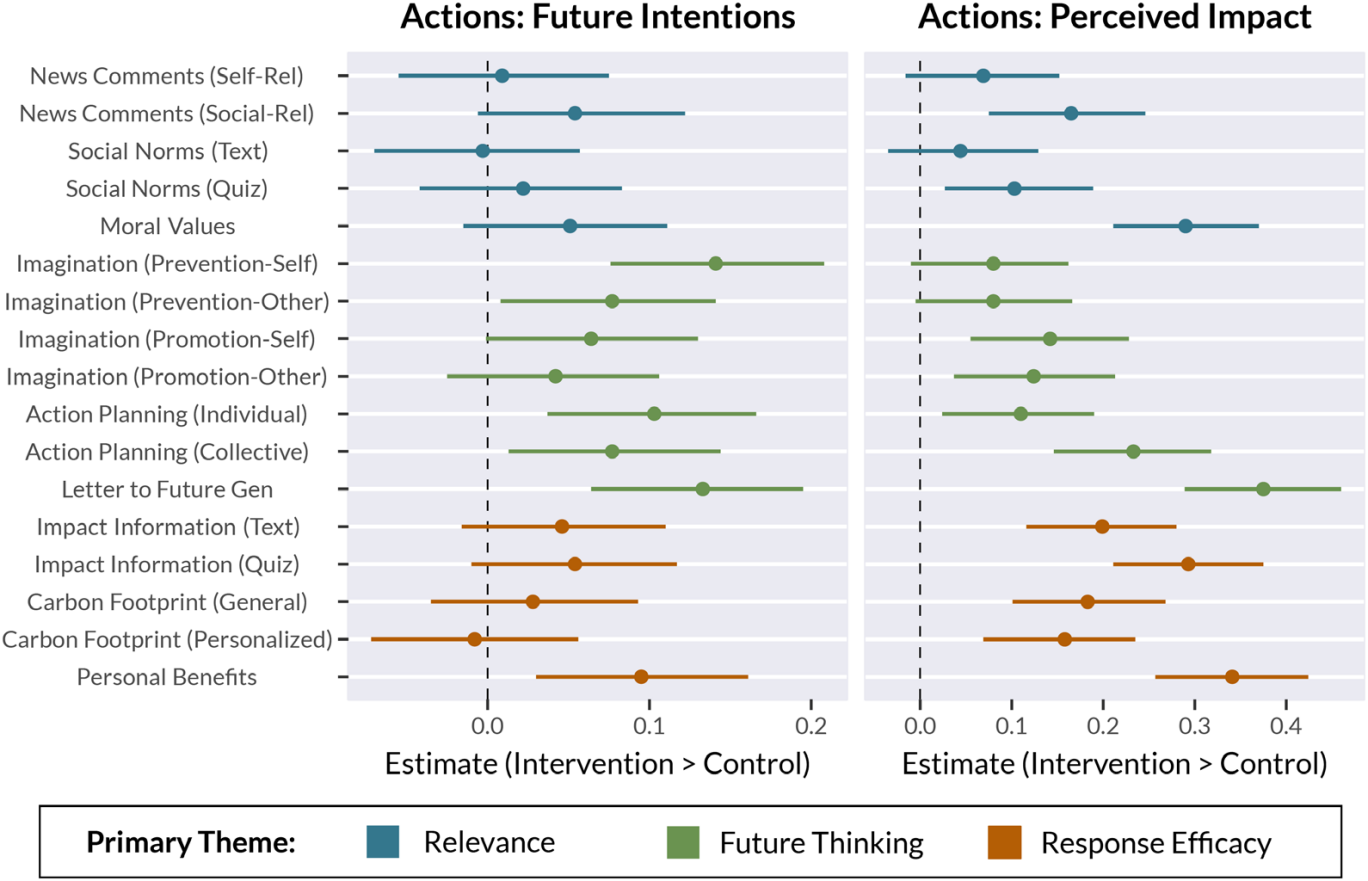
Results from the Climate Action Task, including action intentions (*Left*) and perceived impact of pro-environmental behaviors (*Right*). Results shown are estimates derived from Bayesian mixed-effects regression models. Point estimates indicate the treatment effect for each intervention condition (Intervention–Control, comparing the median values from each posterior distribution). Error bars mark 95% credible intervals surrounding the point estimates. Dependent variables were z-scored to provide standardized effect sizes. Dotted lines marks zero (no effect; no difference from Control group). Points are color-coded to reflect the three intervention themes: Relevance, Future Thinking, and Response Efficacy. Note that some interventions can be described by more than one theme (Figs. 1 and 2); colors here indicate a primary theme for each intervention.

We predicted that the interventions would increase intentions to engage in pro-environmental behaviors, relative to the Control group. Although we expected that all interventions had the potential to motivate action, we predicted that interventions that targeted Future Thinking would be most effective. Consistent with our predictions, several interventions effectively increased action intentions, particularly the interventions that targeted Future Thinking (Fig. 3, *Left* and *SI Appendix*, Table S3). The Prevention-Self variant of the Guided Imagination intervention had the strongest effect on action intentions, closely followed by the Letter to Future Generation intervention. Several other interventions also increased action intentions (in decreasing order of effect size): Action Planning (Individual), Personal Benefits, Guided Imagination (Prevention-Other), and Action Planning (Collective). Overall, results support the idea that imagining future actions and outcomes is an effective strategy for motivating climate action, particularly when combined with appeals to self- and social-relevance. We also explored intentions across categories of actions (e.g., diet-related, transit-related, collective actions); results by category are reported in *SI Appendix*, Table S4. Notably, the two leading interventions—Guided Imagination (Prevention-Self) and Letter to Future Generation—broadly increased intentions to engage in both collective and individual actions.

Our secondary outcome measures offer additional insight into potential mechanisms of action (*SI Appendix*, *Supplementary Results*). The most effective interventions for motivating action evoked high-arousal emotions; the Guided Imagination (Prevention-Self) condition increased anger, fear, and perceived risk, whereas the Letter to Future Generation condition increased anger, hope, and determination. Other effective interventions, such as the Personal Benefits and Action Planning interventions, increased self-efficacy. In contrast, none of the interventions that primarily targeted Future Thinking decreased temporal, geographic, or social aspects of psychological distance associated with climate change, suggesting that the benefits of these interventions were not driven by reducing psychological distance.

Overall, several interventions effectively increased intentions to engage in pro-environmental behaviors, particularly interventions under the Future Thinking theme. The most effective strategies targeted both Future Thinking and Relevance. The benefits of these interventions appeared to be driven by high-arousal emotions and/or increased self-efficacy. Our results also indicate that reducing psychological distance may not be necessary for motivating action.

### Perceived Impact of Pro-Environmental Behaviors.

Next, we investigated whether the interventions increased the perceived impact of pro-environmental behaviors. We expected that interventions under the Response Efficacy theme would have the strongest effects on perceived impact. Participants rated perceived impact for each action after reporting current behavior and future intentions (*Materials and Methods*, Climate Action Task). Most of the interventions (13 of 17 conditions) increased perceived impact relative to the Control group (Fig. 3, *Right* and *SI Appendix*, Table S5). The most effective conditions were the Letter to Future Generation, Personal Benefits, Moral Values, and Impact Information (Quiz) interventions. We also explored whether perceived impact differed across action categories (e.g., diet-related, transit-related, collective actions); results are reported in *SI Appendix*, Table S6.

Notably, all of the interventions in the Response Efficacy theme increased perceived impact, as expected given that these interventions emphasized impact (for the environment or for oneself). However, several interventions belonging to other themes were also effective, suggesting that directly providing information about impact was not necessary to increase perceived impact. Analysis of secondary measures revealed that most of the interventions that increased perceived impact also increased self-efficacy and feelings of determination associated with climate change (*SI Appendix*, *Supplementary Results*).

### Intentions to Share Information about Climate Change.

**Fig. 4.**
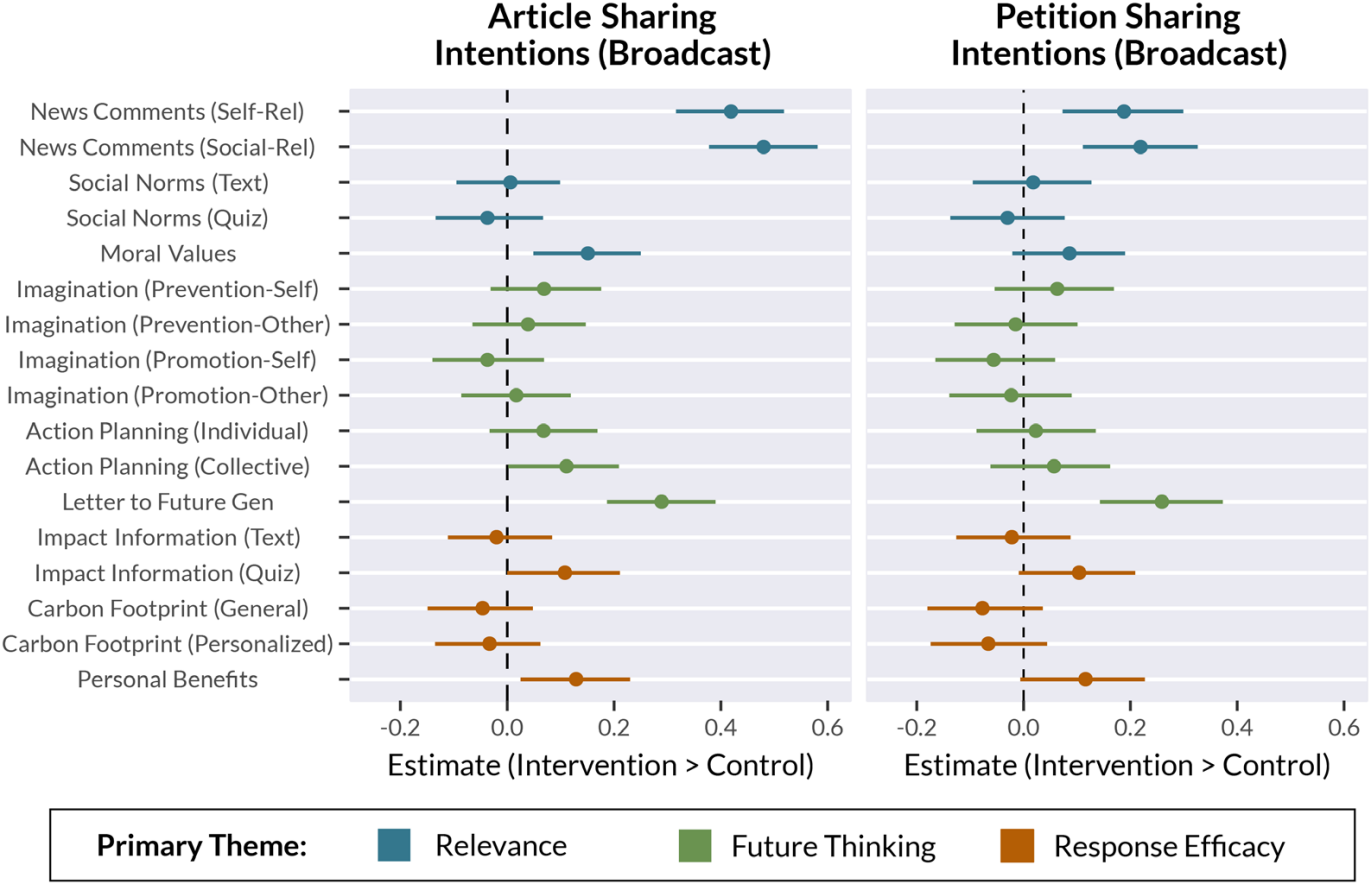
Results for intentions to share news articles (*Top*) and petitions (*Bottom*) about climate change broadly on social media (“broadcast” sharing). Results for narrowcast sharing are visualized in *SI Appendix*, Fig. S2. Results shown are estimates derived from Bayesian mixed-effects regression models. Point estimates indicate the treatment effect for each intervention condition (Intervention–Control, comparing the median values from each posterior distribution). Error bars mark 95% credible intervals surrounding the point estimates. Dependent variables were z-scored to provide standardized effect sizes. Dotted lines marks zero (no effect; no difference from Control group). Points are color-coded to reflect the three intervention themes: Relevance, Future Thinking, and Response Efficacy. Note that some interventions can be described by more than one theme (Figs. 1 and 2); colors here indicate the primary theme for each intervention.

We predicted that the interventions—particularly those under the Relevance theme, which emphasized self- and social-relevance of climate change—would increase intentions to share information about climate change. Results for all information sharing outcomes are visualized in Fig. 4 (broadcast sharing) and Fig. S2 (narrowcast sharing), and reported in *SI Appendix*, Tables S7 (articles) and S8 (petitions).

#### Sharing news headlines.

We first investigated intentions to share news articles about climate change broadly on social media (broadcast sharing). Broadcast sharing intentions for news articles were greatest in the two conditions within the News Comments intervention (Social-Relevance and Self-Relevance). Several other interventions also increased broadcast sharing intentions relative to the Control group (in decreasing order of effect size): the Letter to Future Generation, Moral Values, Personal Benefits, Impact Quiz, and Collective Action Planning conditions all had small effects on broadcast sharing intentions.

Next, we repeated the analysis described above to investigate intentions to share news articles directly with another person (narrowcast sharing). Results were very similar to the analysis of broadcast sharing intentions. Narrowcast sharing intentions were greatest in the Social-Relevance variant of the News Comments intervention, followed by the Self-Relevance variant of the same intervention and the Letter to Future Generation intervention. The Moral Values, Personal Benefits, and Collective Action Planning conditions all had smaller effects on narrowcast sharing intentions.

#### Sharing petitions.

Using the same approach as for the analysis of intentions to share news articles, we then investigated broadcast and narrowcast sharing intentions regarding *petitions* about climate change. Broadcast sharing intentions for petitions were greatest in the Letter to Future Generation intervention and the Social-Relevance variant of the News Comments Intervention, followed by the Self-Relevance variant. The Personal Benefits and Impact Quiz conditions also slightly increased broadcast sharing intentions relative to the Control group.

In a separate model, we assessed narrowcast sharing intentions for petitions. The Letter to Future Generation intervention had the greatest effect on narrowcast sharing intentions, followed by the Social-Relevance variant of the News Comments intervention, the Personal Benefits intervention, and the Self-Relevance variant of the News Comments intervention.

#### Summary of sharing intentions.

Overall, we found that the News Comments interventions (particularly the Social-Relevance variant) and the Letter to Future Generation intervention were broadly effective at increasing intentions to share both news articles and petitions about climate change (Fig. 4, *SI Appendix*, Fig. S2). Although other interventions also had small effects on sharing intentions, these conditions were consistently among the most effective. Our secondary measures offer insight into underlying mechanisms; the News Comments and Letter to Future Generation interventions all increased the perceived self-relevance and social-relevance of news headlines about climate change (*SI Appendix*, *Supplementary Results*). Taken together, these results are consistent with the idea that increasing the perceived self- and social-relevance of information motivates sharing (9, 10, 25).

## Discussion

Psychological interventions have the potential to change behavior at scale to help address climate change. We systematically tested 17 psychological interventions, characterized by three key themes (Fig. 1), which were all expected to engage key neural and psychological systems relevant to behavior change: Relevance (relating climate change to oneself and close others), Future Thinking (imagining future actions and outcomes related to climate change), and Response Efficacy (targeting beliefs about the environmental and personal benefits of climate action). Our primary aims were to identify strategies to increase intentions to engage in pro-environmental behaviors, the perceived impact of those behaviors, and intentions to share information about climate change. Overall, we identified effective interventions for all primary outcomes, and found that interventions that targeted multiple mechanisms (e.g., thinking about future outcomes for oneself or close others) were generally most effective. Notably, the Letter to Future Generation intervention was broadly effective across all primary outcomes, although other interventions (e.g., News Comments, Guided Imagination) had relatively stronger effects for specific outcomes.

### Motivating Pro-Environmental Behaviors.

We first investigated whether the interventions increased intentions to engage in pro-environmental behaviors, including individual actions (e.g., driving less, eating vegetarian meals, paying for renewable energy to power one’s home) and collective actions (e.g., donating, volunteering, contacting representatives). We found that engaging in future thinking—especially self- and socially focused future thinking—effectively motivated climate action. Six intervention conditions significantly increased intentions to engage in pro-environmental behaviors; all six involved future thinking. The most effective intervention was the Prevention-Self variant of the Guided Imagination intervention, which involved imagining oneself experiencing a preventable negative future scenario due to climate change. Another leading intervention was the Letter to Future Generation condition, which emphasized future outcomes for a socially close child. Other future thinking interventions, such as engaging in action planning or brainstorming near-future personal benefits, also motivated action.

Our secondary outcome measures offer insight into potential mechanisms of action. Perceived risk has previously been linked to climate action (94, 95); the two interventions that increased perceived risk also increased action intentions (Guided Imagination, Prevention-Self; Action Planning, Individual). Several major theories of behavior change, such as The Theory of Planned Behavior (67, 96) and Social Cognitive Theory (92), propose that self-efficacy—beliefs about one’s ability to take action effectively—drives motivated behavior. The two interventions that led to the greatest increases in self-efficacy (Letter to Future Generation and Personal Benefits) also increased action intentions. Future thinking interventions also modulated emotions about climate change, such as by evoking anger (Guided Imagination, Prevention-Self; Letter to Future Generation); anger is a high-arousal emotion associated with “approach” motivation, which can catalyze action (97). Taken together, these findings suggest that engaging in self- and socially relevant future thinking may motivate action via several distinct mechanisms, such as by increasing perceived risk, self-efficacy, or anger.

Contrary to our expectations, the leading future thinking interventions did not decrease any aspects of psychological distance related to climate change, suggesting that the benefits of future thinking were not driven by reducing psychological distance. These results contribute to an ongoing theoretical debate about the importance of psychological distance in climate change interventions; our findings align with recent evidence that psychological distance may be overestimated and not always related to action intentions (59–61).

### Increasing Perceived Impact.

We also tested whether the interventions increased the perceived impact of pro-environmental behaviors. Our measure of perceived impact assessed outcome expectancies, a key factor identified in major theories of behavior change, such as Social Cognitive Theory (92). We prompted participants to rate how much each action would help to reduce the negative effects of climate change if many people engaged in the action. This measure probes collective response efficacy (i.e., expected positive outcomes resulting from many people taking action), which prior work has theorized may be important for motivating action to address large-scale societal problems like climate change (75, 98).

Most of the interventions tested in our tournament (76.5%, 13/17 interventions) increased the perceived impact of pro-environmental behaviors. All interventions in the Response Efficacy theme increased perceived impact; interestingly, even emphasizing personal impact (Personal Benefits condition) also increased perceived environmental impact. Overall, the Letter to Future Generation, Personal Benefits, Impact Quiz, and Moral Values interventions led to the greatest increases in perceived impact.

Interestingly, results for perceived impact were distinct from results for action intentions. For instance, the Carbon Footprint and Impact Information interventions, which both directly provided information about the mitigation potential of pro-environmental behaviors, substantially increased perceived impact but did not increase action intentions. Conversely, the leading strategy for motivating action (Guided Imagination, Prevention-Self) did not increase perceived impact. These results offer theoretical implications, suggesting that although beliefs about impact and efficacy are often correlated with behavioral intentions (92), changing these beliefs may not be necessary or sufficient for motivating climate action. It is also important to note that perceived impact is poorly aligned with actual impact (79–81); individuals tend to overestimate the mitigation potential of actions like recycling, and underestimate the potential of actions like reducing driving. In related work, we are investigating multipart intervention strategies that aim to both correct misconceptions about impact and motivate action, directing effort toward the actions that matter most.

### Motivating Information Sharing.

We also investigated whether the interventions increased intentions to share news articles and petitions about climate change. For each article and petition, participants rated their willingness to share the content broadly on social media or directly with someone they know.

The two variants of the News Comments intervention, in which participants wrote comments identifying why news headlines about climate change were relevant to themselves or close others, had the strongest effects on intentions to share the news headlines. These results replicate our prior work, adding to the extensive body of evidence indicating that perceived self- and social-relevance of information drives sharing (10, 11, 22–25). Extending prior studies, we also found that the effects of the News Comments interventions generalized, increasing intentions to share petitions during a subsequent task (i.e., without writing comments about the petitions).

A recent global megastudy, which also used an intervention tournament approach, found that the most effective strategy for motivating individuals to share information about climate change on social media was negative emotion induction, which led to 12% greater sharing intentions relative to the control group (4). However, this intervention also had a robust backfire effect on pro-environmental behavior. We also assessed broadcast sharing intentions with a comparable rating scale; our leading intervention (News Comments, Social-Relevance) had a strong effect (16% increase in sharing intentions, relative to the Control group) and did not decrease action intentions.

The Letter to Future Generation intervention, in which participants wrote a letter about climate change to a socially close child (as if the letter would be delivered in the future), also substantially increased intentions to share news articles and petitions. Our results conceptually replicate recent evidence that this intervention strategy motivated information sharing on social media (4); we extend prior findings by demonstrating this effect with multiple real news articles and petitions about climate change. Several other interventions that appealed to self-relevance (Moral Values, Personal Benefits), also had small effects on sharing intentions.

Overall, interventions that appealed to self- and social-relevance were the most effective for motivating people to share information about climate change. The leading interventions for motivating information sharing (News Comments and Letter to Future Generation) also increased the perceived self- and social-relevance of climate-related news, consistent with the idea that perceived relevance is a mechanism driving intentions to share information (10, 11, 22–25).

### Tournament Insights: Assessing Relative Effectiveness.

The urgency and global scale of climate change underscore the importance of identifying the *most effective* strategies for changing behavior. An intervention tournament approach, in which many strategies are systematically tested and compared, is ideal for addressing this challenge. Intervention tournaments allow researchers to test competing hypotheses from distinct theoretical frameworks and identify the most effective strategies. In contrast to independent studies, in which results may be attributed to different samples, recruitment methods, tasks, outcome measures, statistical analysis, location, or time of year, a tournament approach enables clear comparison across interventions.

Crucially, in addition to identifying the most effective strategies for each goal, we also identified *ineffective* strategies. For example, interventions that provide feedback about individuals’ carbon footprints are widely promoted by major environmental agencies, such as the U.S. Environmental Protection Agency (82) and the World Wildlife Fund (83). Despite the popularity of such tools—first developed and promoted by British Petroleum (84)—there is little empirical evidence of effectiveness (85, 86). We demonstrate that this prevalent climate communication strategy failed to motivate behavior change. Our results identify alternative, more effective communication strategies that should be prioritized over carbon footprint information.

Our results also offer insights and generate new questions pertaining to underlying psychological mechanisms. Behavioral interventions can operate via multiple distinct mechanisms, and the relative contributions of these mechanisms can differ across contexts (99, 100). For instance, hope is positively related to climate action and policy support, but these effects vary across contexts and depend on both cognitive (e.g., future thinking) and emotional mechanisms (101). The leading interventions identified in our tournament targeted multiple mechanisms (e.g., Letter to Future Generation), suggesting that interventions that employ an ensemble of strategies to target multiple mechanisms may be more effective. Future work testing the effectiveness of multipart interventions that target multiple mechanisms by combining elements of the leading interventions identified in our tournament would be fruitful.

Our findings complement and extend insights from a recent cross-cultural study that also used an intervention tournament approach (4). This recent study tested eleven interventions across 63 countries, identifying several promising strategies for increasing belief in climate change, policy support, and information sharing intentions. However, none of the interventions tested in the prior tournament increased climate action (operationalized as completing math worksheets in exchange for donations to a tree planting organization), and several of the interventions decreased action. A strength of the previous climate action task was the direct measurement of effortful behavior, but a limitation is that it did not direct participants toward actions that they could repeatedly take in everyday life to mitigate climate change. Notably, in the prior study, some of the most effective interventions for one outcome (e.g., information sharing) had robust backfire effects on climate action.

Our study builds on valuable insights from this other recent intervention tournament (4) in several ways. We tested a different set of interventions, selected and classified to target key psychological and neural mechanisms; in contrast, the prior tournament crowd-sourced intervention ideas from the research community. Importantly, we also included a more extensive set of measures, investigating distinct outcomes of interest (e.g., perceived impact of climate action, intentions to share news and petitions, petition signing) and underlying mechanisms (e.g., self-efficacy beliefs, perceived risk, emotions, psychological distance). Our approach thus offers insight into underlying mechanisms, considers how each intervention acts upon one or more of these mechanisms, and reveals converging evidence by testing multiple intervention strategies under each mechanistic theme. We also identified promising interventions that differ from this prior tournament, including several strategies that effectively motivated action and strategies that motivated information sharing without backfiring on action intentions.

### Limitations and Future Directions.

One limitation of the present study is that we measured behavioral intentions as opposed to directly observable behavior. Behavioral intentions are reliably related to actual behavior (67, 96, 102), but other factors (e.g., effort, cost, forgetting) may prevent individuals from acting on their intentions. An important goal for future research is to test whether our leading interventions effectively change real-world behavior over time, particularly by using objectively logged or observable measures. Future studies could test the leading interventions identified in our tournament with direct measures of effortful behavior (e.g., donations to environmental organizations, signing up for home renewable energy programs) and longitudinal measures (e.g., using ecological momentary assessments).

A recent critique of psychological interventions to address societal challenges like climate change is that such interventions focus on individuals (“i-frame”), potentially diverting attention and support away from systemic change (“s-frame”) (103). We argue that both individual- and systemic-level changes are necessary to address climate change, and that these frames are neither independent nor in opposition (14, 104, 105). Collective action arises from the coordinated actions of individuals; policy changes influence how individuals perceive issues and social norms; individuals elect, contact, and lobby representatives to shape policy (14, 104–106). We observed that several of our interventions broadly increased intentions to engage in individual *and* collective actions to address climate change, suggesting that some interventions can increase support for both forms of climate action. However, it is also important to note that the Carbon Footprint interventions—an “i-frame” approach that is currently widely used—failed to motivate behavior change, underscoring the importance of identifying and implementing effective strategies to motivate both individual and collective actions.

It is also worth noting that we observed small-to-medium effect sizes for leading interventions across outcome measures. However, even small effects can have substantial impact at scale: brief online interventions can be distributed to large audiences, individuals engage in actions habitually in daily life, and the effects of sharing information spread through social networks (107, 108). In addition, we focused on a limited set of pro-environmental behaviors that we identified to be feasible for individuals and relatively high-impact in terms of potential to mitigate carbon emissions. Future research could also explore strategies to motivate pro-environmental behaviors in other social roles or contexts, such as in workplaces, schools, and community organizations.

In our sample, we aimed to approximate the demographic diversity of the United States in terms of age, race, and gender (see *SI Appendix*, Table S1). However, there are several limitations: we did not investigate cross-cultural differences, Hispanic/Latino participants were underrepresented in our sample, and we lack sufficient statistical power to investigate demographic differences across conditions. Building on recent cross-cultural research (4), future studies could also test the effectiveness of our interventions globally.

In addition, the distribution of political ideology was not nationally representative; our sample included more liberals than conservatives (*SI Appendix*, Fig. S1). Climate change is a politically polarized issue—in the United States and globally, conservatives are less likely to believe in climate change, perceive climate change as a threat, and support action to address climate change (109, 110). During initial recruitment and preprocessing, we also excluded participants who reported denying the existence or anthropogenic causes of climate change. Although only 13% of Americans do not believe that climate change is not occurring (111), a larger subset of the population is uncertain about the anthropogenic causes of climate change. An important goal for future research is to identify strategies that are effective for individuals who hold doubtful or dismissive beliefs related to climate change. In ongoing work, we are investigating strategies to bridge the partisan divide and replicating promising interventions in politically balanced samples that include individuals who are skeptical about the causes and impacts of climate change.

## Conclusion

Results from our tournament offer actionable insights for scalable behavioral interventions and climate communication. We found that the most effective strategies to motivate action to address climate change involved guiding people to think about future outcomes, particularly for themselves and close others. Reflecting on social relevance (relating climate change to people you know) was the most effective strategy to motivate people to share news articles and petitions about climate change. Our findings are broadly relevant to psychological theories of behavior change, motivation, social behavior, decision making, learning, and information sharing.

Our findings also offer practical and actionable implications for communicators, policymakers, and environmental scientists. Importantly, the promising interventions identified in our tournament could be adapted to create accessible, engaging, and interactive online tools. In future work, we aim to scale our leading interventions to such online platforms; we have previously developed and disseminated similar tools to reach millions of users (112, 113). In ongoing work, we are laying foundations to implement our leading interventions through displays and interactive activities in museums, and partnering with environmental journalists to apply insights from our tournament to broader climate communication. Overall, we recommend illustrating future scenarios and emphasizing the personal and social impact of climate change as leading strategies to promote behavior change and information sharing.

## Materials and Methods

### Participants.

Detailed information about the sampling procedure, power analyses, and demographics are reported in *SI Appendix*. The study was approved by the Institutional Review Board at the University of Pennsylvania (protocol #854102). In brief, we recruited online paid participants through Prolific who were U.S. residents, fluent in English, ages 18+, had high prior task approval ratings, and reported believing in climate change. We used quota sampling to stratify our sample by gender and age group, recruiting participants across the adult lifespan (ages 18 to 88). Participants provided informed consent by clicking a button at the start of the task. Participants were compensated with $5 for a study that took approximately 25 min to complete (a rate of approximately $12/h).

Data collection took place in two phases. In the first phase of data collection (February 2024), we tested six overarching intervention strategies. To determine the most effective implementation of each intervention strategy, we also tested multiple variations within each “parent” intervention. Participants were randomly assigned to one of fourteen intervention groups or the no-intervention Control group. In the second phase of data collection (June 2024), we tested three additional late-breaking intervention ideas (without variations). We pooled data from the two samples to compare results from all interventions (9 broad intervention strategies, 17 intervention groups in total) with the same Control group. After exclusions (*SI Appendix*, *Exclusions*), the final sample included 7,624 participants (6,443 in sample 1; 1,181 in sample 2). Demographic information is provided in *SI Appendix*, Table S1.

### Procedure.

Below, we briefly describe each intervention task, grouped by the three key themes: *Relevance*, *Future Thinking*, and *Response Efficacy*. Note that some interventions can be described by multiple themes (Fig. 1); for simplicity, below we group interventions by primary themes. Additional methodological details are provided in *SI Appendix*, *Procedure*.

#### Relevance theme.

In the News Comments interventions, participants viewed and wrote brief comments about news headlines related to climate change. These interventions were based on prior evidence that reflecting on the self- and social-relevance of information motivates sharing (10, 22, 23, 25). In the Self-Relevance condition (N = 396), participants described why the headlines mattered to them; in the Social-Relevance condition (N = 392), participants described why the headlines mattered to people they know.

In the Social Norm Information interventions, participants viewed statistics from recent U.S. national polls, describing normative attitudes about climate change (e.g., policy support, climate change denial). These interventions were based on evidence that people tend to underestimate normative belief and concern about climate change, and changing perceived social norms could motivate action (15, 19, 20, 114). In the Norm Quiz condition (N = 426), participants guessed a missing statistic before the correct answer was revealed; in the Norm Text condition (N = 428), participants viewed intact statements.

In the Moral Values intervention (N = 420), participants read brief descriptions of six moral values adapted from Moral Foundations Theory (115, 116). This intervention was based on evidence that relating climate change to one’s moral values could change attitudes (117). Participants selected the moral value that was most important to them, completed a writing exercise, and read a persuasive message that related their chosen moral value to climate change.

#### Future thinking theme.

In the Guided Imagination interventions, participants completed a structured imagination exercise. These interventions were based on evidence that engaging in *episodic simulation* (i.e., imagining hypothetical or future scenarios) can motivate pro-environmental behaviors (53, 118) and change beliefs about risk (51, 52, 119). In the Prevention-Self condition (N = 380), participants imagined themself experiencing a preventable negative future that could occur due to climate change. In the Promotion-Self condition (N = 373), participants imagined themself experiencing a positive future that could arise from action to address climate change. In the Prevention-Other (N = 374) and Promotion-Other (N = 374) conditions, participants imagined a fictional character in the same negative and positive future scenarios, respectively.

In the Action Planning interventions, participants developed a plan to achieve a goal and imagined the process. These interventions were adapted from *Mental Contrasting with Implementation Intentions* tasks, which have been shown to motivate behavior change (120–123). Participants selected an action from a list of recommended actions to mitigate climate change, then imagined and described the process of engaging in the action, potential obstacles and solutions, and eventual outcomes. In the Individual Action Planning condition (N = 393), participants selected an individual action goal (e.g., taking a train instead of flying, eating less red meat), whereas in the Collective Action Planning condition (N = 382) they selected a collective action goal (e.g., donating, volunteering, contacting representatives).

In the Letter to Future Generation intervention (N = 391), participants identified, described, and wrote a brief letter to a child or teenager they personally knew. This intervention was adapted from a task that was previously shown to increase climate-related policy support and information sharing (92). Participants imagined that their letter would be delivered in the year 2050, when the child would be an adult. In the letter participants were asked to tell the child about their personal efforts to address environmental problems with the goal of ensuring that the child would inherit a habitable planet.

#### Response efficacy theme.

In the Impact Information interventions, participants learned about the impact (in terms of mitigating greenhouse gas emissions) of actions that individuals could take to help mitigate climate change. These interventions were based on evidence that surprising feedback can correct misconceptions (124–126). In the Impact Quiz condition (N = 416), participants guessed the values before impact estimates were revealed; in the Impact Text condition (N = 418), participants viewed intact statements.

In the Carbon Footprint interventions, participants learned about actions that they could take to reduce their carbon footprints. These interventions were included in the tournament because carbon footprint estimators are widely used and promoted by organizations like the Environmental Protection Agency (82) and World Wildlife Fund (83), despite limited evidence of effectiveness. In the Personalized Carbon Footprint condition (N = 413), participants received personalized feedback about their current carbon footprint and how various actions would reduce it. In the General Carbon Footprint condition (N = 428), participants received feedback calculated for an average U.S. resident.

In the Personal Benefits intervention (N = 370), participants generated short-term *personal* benefits (e.g., improving health, happiness, or finances) that could result from engaging in pro-environmental behaviors. This intervention was based on evidence that people tend to value short-term rewards over long-term outcomes (127), and that positive attitudes toward behaviors (67) and short-term rewards increase goal pursuit (46–48). For each action, participants brainstormed as many personal benefits as possible (text entry), thinking of the effects of engaging in the action over the next six months.

#### Outcome measures.

After completing an intervention task (or after consent in the Control group), participants completed the Climate Action, News Headlines, and Petitions Tasks (described below) in a randomized order. In the News Comments interventions, however, participants always completed the News Headlines task first, because these interventions modified this task by adding a writing component. After the primary tasks, participants completed a series of secondary measures in a randomized order. In addition to the measures described below, we collected additional measures for exploratory analyses. Additional information is provided in *SI Appendix*.

##### Climate action task.

Participants were asked about 12 actions that could have positive or negative effects on climate change, including individual actions (e.g., eating vegan meals, flying by airplane, paying for renewable energy to power one’s home) and collective actions (e.g., donating, volunteering, or contacting representatives). In a pilot study, we assessed beliefs about pro-environmental behaviors, identifying actions that were feasible but not yet widely adopted. From this list of actions, we selected a subset of target actions that were associated with relatively greater reduction of greenhouse gas emissions (93).

Actions were presented in a randomized order, with a single action per page. Participants reported their current frequency of engaging in each action (e.g., typical driving habits, annual donations to environmental organizations), using custom scales for each action. Participants then used 7-point Likert scales to rate their intentions to engage in the action more/less in the future (1 = *A lot less*, 7 = *A lot more*) and the perceived environmental impact if many people did the action more/less often (1 = *No impact*, 7 = *Very large impact*). Actions were framed in terms of engaging “more” or “less” depending on which direction would indicate pro-environmental behavior (e.g., driving *less*, donating *more*).

##### News headlines task.

Participants viewed a set of five news headlines about climate change (consisting of a title and an accompanying lede), randomly selected from a larger set of 26 headlines sourced from the New York Times. For each article presented, participants used a scale from 0 (*strongly disagree*) to 100 (*strongly agree*) to rate their intentions to share the article broadly on social media (broadcast sharing) or directly with someone they know (narrowcast sharing). Using the same rating scales, participants also rated the perceived self-relevance and social-relevance of each news article.

##### Petitions task.

Participants viewed three petitions about climate change (screenshots of real petitions from *change.org* accompanied by abbreviated text), randomly selected from a larger set of 10 petitions. For each petition presented, participants used a scale from 0 (*strongly disagree*) to 100 (*strongly agree*) to rate broadcast sharing intentions, narrowcast sharing intentions, and intentions to sign the petition. Participants also had the option to click a link to view the petition and sign it; however, due to a programmatic error, not all click-tracking data were saved.

##### Secondary measures.

Secondary measures for exploratory analyses included scales assessing self-efficacy, perceived risk, emotions, psychological distance, self-reported knowledge, and uncertainty/skepticism regarding climate change. Participants also completed a standard demographics survey.

### Statistical Analysis.

#### Open science practices.

Data, code, and fitted Bayesian models are publicly available in a permanent repository hosted by the Open Science Framework (https://doi.org/10.17605/OSF.IO/X9C6J) (128). Overall analyses of the entire tournament sample were not preregistered. However, we preregistered the methods and predictions for most individual interventions; these preregistrations include some additional condition-specific analyses that are beyond the scope of this report (https://osf.io/x9c6j/registrations) and will be handled in individual intervention-specific papers. Survey/task materials and additional information about standard operating procedures can also be found within the project repository.

#### Statistical modeling.

Analyses were conducted in R (version 4.4.1), implemented with RStudio (version 2024.04.2). We used Bayesian analyses to estimate intervention effects for each outcome measure, comparing each intervention group with the Control group. We used a Bayesian approach because the goal of the study was to estimate the effectiveness of each intervention approach, focusing on effect magnitude rather than the presence or absence of an effect. We report results with point estimates (median of posterior distribution) for each group and the 95% credible interval. We interpret effects as significantly different from the Control group if the lower bound of the 95% credible interval is greater than the Control group point estimate. For all analyses, we used weakly informative priors (Gaussian distribution with *M* = 0, SD = 1). We used linear mixed-effects regression models (for tasks with multiple observations per participant) and linear regression models (for tasks with single observations or composite scores). For measures of current action frequency from the Climate Action Task, we z-scored values within-item to account for discrepancies in scale (e.g., dollars donated vs. miles driven), then included this standardized current frequency variable in statistical models as a covariate. Additional information about random effects specification, software packages, and data cleaning is provided in *SI Appendix*, *Statistical Analysis*.

## Supplementary Material

Appendix 01 (PDF)

## Data Availability

Anonymized (Data and code needed to reproduce results) data have been deposited in Open Science Framework (https://doi.org/10.17605/OSF.IO/X9C6J) (128).
