# Supplementary material for: Behavioral interventions motivate action to address climate change
Source: Proc Natl Acad Sci U S A. 2025 May 13;122(20):e2426768122. doi: 10.1073/pnas.2426768122 (PMC12107114; doi:10.1073/pnas.2426768122)
Supplement: Supplementary file 1 — Appendix 01 (PDF) [file pnas.2426768122.sapp.pdf]

PNAS

Supporting Information for
Behavioral Interventions Motivate Action to Address Climate Change

Alyssa H. Sinclair, Danielle Cosme, Kirsten Lydic, Diego A. Reinero, José Carreras-Tartak, Michael E. Mann, & Emily B. Falk

Corresponding Authors: Alyssa Sinclair, Emily Falk, Michael Mann
Emails: asinclair@falklab.org, emilybfalk@falklab.org, mmann00@sas.upenn.edu

This PDF file includes:

Supplementary Methods
Supplementary Results
Tables S1 to S16
Figures S1 to S2
SI References

Supplementary Methods

Participants

Sampling Procedure

The sample size was determined *a priori* by conducting power analyses. We determined that a sample size of 400 participants per condition would yield sufficient power to detect effects of interest. For the News Headline Task (with five repeated measures per participant), this sample size provided >87% power to detect between-group differences for effects sized Cohen's d = 0.15 or larger. For the Petition Signing Task (with three repeated measures per participant), this sample size provided >81% power to detect between-group differences for effects sized d = 0.15 or larger. For the Climate Action Task (with twelve repeated measures per participant), this sample size provided >92% power to detect between-group differences for effects sized d = 0.15 or larger. This sample size also provided 80% power to detect between-group differences in secondary outcomes (single measure per person) sized d = 0.2 or larger. In anticipation of exclusions, we aimed to recruit additional participants to increase the sample in each condition by 5% (20 additional participants per intervention group and 40 additional participants in the Control group). Participants were randomly assigned to either an intervention condition or the Control group in a between-subjects design.

Participants were recruited from Prolific, an online platform for paid study participation. The initial sample (sample 1) was collected during February 2024. Inclusion criteria were as follows: currently residing in the United States, fluent in English, at least 18 years of age, at least a 95% prior approval rating, prior completion of at least 50 submissions, and belief in climate change (response to a single-item Prolific pre-screening question, "Do you believe in climate change?"). We used quota sampling to recruit evenly by gender (50% men, 50% women); these binary categories are used by Prolific for gender-balanced samples, but we allowed participants to report non-binary identities in our custom demographics survey. We also used quota sampling to recruit participants across the adult lifespan (40% younger adults aged 18-35, 40% middle-aged adults aged 36-54, and 20% older adults aged 55-90). Younger and middle-aged adults were oversampled due to the skewed age distribution on Prolific; less than 10% of the platform participants are aged 55-90. In the first phase of data collection, once recruitment slowed to fewer than 15 participants per day for middle-aged and older adults (17 days after data collection began), we removed quota sampling by age to allow for the full sample size to be collected. The final 797 participants of the sample (prior to exclusions) were collected without age quota sampling.

The second sample (sample 2) took place during June 2024. During this second phase, we tested an additional three late-breaking interventions using the same sample sizes and procedures as in the first phase. The same inclusion criteria and age/gender quota sampling from the first phase were applied. Participants who had completed a task in sample 1 were not eligible to participate in sample 2 tasks. Once recruitment slowed to less than one participant per day for older adults, we removed quota sampling by gender. The final 16 participants from this sample (prior to exclusions) were recruited without the gender quota. Data from Samples 1 and 2 were combined for analysis.

Exclusions

Participants were excluded if they failed both of the attention checks within the survey (n sample 1 = 15, n sample 2 = 0); the attention check required responding as instructed ("If you are paying attention, select 'somewhat agree' below"). We also excluded participants who reported denying anthropogenic climate change is occurring, either by selecting "I think there is no such thing as climate change" or "Entirely natural processes" on the *Cause of Climate Change* scale, or having a mean > 4 (max score = 5) on the *Uncertainty and Skepticism* scale (n sample 1 = 83, n sample 2 = 11).

To ensure that participants were engaged in the task and not using outside tools to complete their writing prompts (e.g., ChatGPT), we used *TaskMaster*, a Qualtrics-integrated mouse-tracking tool, to identify off-task behavior that may indicate cheating or distraction (1). Participants were informed about

the use of mouse-tracking software and warned not to use external aids during the task. For interventions with multi-page writing components (Action Planning and Guided Imagination interventions), we excluded participants who clicked away from the experiment on every page with writing prompts (*n* sample 1 = 58, *n* sample 2 = N/A).

We additionally excluded participants who provided poor-quality responses to intervention tasks with open-ended writing components, defined as one or more of: entering random text that was not related to the prompt, copying the prompt or headline/snippet text, entering the same text for every prompt, or entering text that does not make logical or grammatical sense (e.g. "I have nothing to say about this", "N/A i Recycle right", "I think this pukes me so much"). These text quality assurances were performed using the large language model GPT-4. For each participant, we prompted the model with instructions, the writing prompts, and the participants' responses. We first tested the validity of the ratings from GPT-4 by manually coding data from 300 participants, evenly sampled from each condition with a writing component. For each written response, GPT-4 and human raters assigned a holistic quality score ranging from 1 (very low quality) to 10 (very high quality), considering the length, relevance, detail, and grammar of the responses. Using these continuous quality scores, we calculated the inter-rater intra-class correlation coefficient between GPT-4 and human ratings to assess reliability within each condition; ICC scores indicated "moderate" to "substantial" agreement (Guided Imagination ICC = 0.74, News Comments ICC = 0.49, Action Planning ICC = 0.46). Having validated this approach, we then used GPT-4 to screen for low-quality responses in the full dataset. We manually reviewed all responses that were assigned low quality scores (≤3 for the Guided Imagination and Action Planning tasks, ≤2 for the News Comments tasks), as well as outlier responses with unusually high or low word count and identified participants to exclude (*n* sample 1 = 51, *n* sample 2 = 49).

At the end of the survey, we also asked participants to self-report dishonesty, distraction, or other issues experienced during the task. Participants were excluded from analysis if they reported using external aids (e.g., Google or ChatGPT) to complete the task, or if they reported answering dishonestly or not taking the survey seriously (*n* sample 1 = 32, *n* sample 2 = 7).

We also excluded participants who failed more than one criterion (*n* sample 1 = 13, *n* sample 2 = 2). After all exclusions, the final combined sample included 7,624 participants (*n* sample 1 = 6,443; *n* sample 2 = 1,181), with 850 participants in the control group and an average of 398 participants per intervention group (range: 370–428). Demographics are reported in Table S1.

Procedure

Overview

We recruited U.S. residents from Prolific, an online marketplace for study participation. After exclusions (see above), the final sample consisted of 7,624 participants across the adult lifespan (ages 18-88 years). Overall, the tournament included nine intervention approaches, spanning three key themes: *Future Thinking, Self- and Social-Relevance*, and *Action Impact* (Figure 1, main text). Although some of these interventions can be described by multiple themes (Figure 1, main text), each intervention is associated with a primary theme (indicated by the leftmost color bar next to each intervention as depicted in Figure 2, main text).

Within some of the nine interventions, we tested multiple conditions (e.g., presenting the same information with plain text vs. an interactive quiz). In total, there were 17 intervention conditions; participants were assigned to one of these groups or a no-intervention control group. In sample 1, participants were randomly assigned to either the control group or a group within one of six interventions (Guided Imagination, Action Planning, News Comments, Social Norm Information, Impact Information, or Carbon Footprints). In sample 2, participants were randomly assigned to either the Letter to Future Generation, Moral Values, or Personal Benefits interventions. Data from both samples were pooled for analysis, enabling all interventions to be compared with the same control group. Below, we describe each of the intervention conditions, grouped by primary theme (Future Thinking, Self- and Social-Relevance, and Action Impact).

Future Thinking Theme

The following interventions promote *future thinking*, such as by encouraging participants to imagine future actions and future outcomes that could impact themselves and others.

Guided Imagination. In the Guided Imagination intervention conditions, participants completed one of four different guided imagination exercises. In each of these exercises, participants were prompted to use all of their senses to simulate a future scenario. We varied the emotional framing of the scenario (Prevention vs. Promotion) and whether participants imagined themself or a hypothetical future person experiencing the scenario (Self vs. Other). All scenarios were closely matched in terms of content and length. These scenarios are briefly described below. The primary theme for these interventions was Future Thinking; the Self variants of the task were also included under the Self- and Social-Relevance theme (Figure 1, main text).

In the *Prevention-Self* condition (n = 380), participants were instructed to envision "a negative future that awaits us if we fail to take action against climate change." At the start of the imagination exercise, participants were prompted, "Imagine that you are walking through the streets of a city that has been transformed by climate change. The sun beats down relentlessly, and the air is thick with smog and orange haze that makes it hard to see. There is a haze above the pavement distorting the air. Close your eyes and visualize this city." After the imagination phase, participants opened their eyes and typed in a textbox to describe what they imagined. This process repeated with three additional prompts extending the scenario. After establishing the context of the city, participants imagined themself walking down the sidewalk in the city. Participants were then instructed to use all of their senses (e.g., sight, sound, smell, touch) to imagine being immersed in the scene, surrounded by people and cars amidst the heat and smog. Lastly, participants were asked to imagine and describe the emotions they would feel in this scenario. In the *Prevention-Other* condition (n = 374), participants imagined the same scenario, but were instructed to imagine a future person who lived in the city experiencing the scenario.

In the *Promotion-Self* condition (n = 373), participants were instructed to envision "a positive future that awaits us if we take action against climate change." Paralleling the Prevention scenario, participants were first instructed, "Imagine that you are walking through the streets of a futuristic city that has taken action to become sustainable. The streets and rooftops are full of greenery, and the skyscrapers are adorned with solar panels. The sidewalks are clean and shaded by trees. Close your eyes and visualize this city." As in the Prevention conditions, participants repeated the process of reading a prompt, imagining with eyes closed, and then typing to describe what they imagined. In response to subsequent prompts, participants imagined walking through a park in the city, then used all their senses to imagine being immersed in the scene, surrounded by pedestrians, bikes, and electric cars. Lastly, participants imagined the emotions they would feel in this scenario. In the *Promotion-Other* condition (n = 374), participants imagined the same scenario, but were instructed to imagine a future person who lived in the city experiencing the scenario.

To ensure engagement with the task, participants were required to provide a written response to each imagination prompt, and were not permitted to move on to the next page of the task until at least seven seconds had elapsed. As in other interventions with writing components, we warned participants not to use external aids (e.g., AI tools for text generation) and used mouse-tracking software to identify and exclude participants who consistently showed off-task behavior on each page of the writing task.

Action Planning. In the Action Planning intervention conditions, adapted from *Mental Contrasting with Implementation Intentions* tasks (2–5), participants completed one of two guided imagination and planning exercises regarding a pro-environmental behavior that one could engage in. The primary theme for these interventions was Future Thinking, but these interventions are also included under the Self- and Social-Relevance and Action Impact themes; the tasks involve thinking about the impact of your actions for yourself, your community, and the environment (Figure 1, main text).

In each exercise, participants first selected a target climate action behavior that they could most easily see themselves doing more than they currently did. In the *Individual Action Planning* condition (n = 393), participants selected an individual action behavior from the following list: driving less, flying less, paying for green energy at home, eating less red meat, or eating more vegetarian or vegan meals. In the *Collective Action Planning* condition (n = 382), participants selected a collective action behavior from the

following list: contacting representatives about climate change, having conversations about climate change, donating to environmental organizations or campaigns, signing climate change related petitions, or volunteering for environmental organizations or campaigns.

In both conditions, participants were asked to consider it a personal goal to engage in the selected action, and to clearly imagine a future in which they take this action. Participants were asked to respond to a series of writing prompts to guide this imagination. First, participants were asked to imagine engaging in the action, including how, when, where, and with whom they would engage in the action. Secondly, participants were asked to imagine the best possible outcomes of the action for themselves, their community, and the environment. Next, participants were asked to concretely imagine the steps necessary to begin engaging in the action, and to map out the action sequence. Finally, participants were asked to consider the biggest obstacle that could prevent them from engaging in the action, and to develop an if-then plan for overcoming this obstacle. At the end of the exercise, participants were asked to review and read aloud an overview of their responses to each prompt. Written responses to each prompt were required to ensure engagement. As in other interventions with writing components, participants were asked not to use external aids, and mouse-tracking software was used to exclude participants who showed consistent off-task behavior.

Letter to Future Generation. In the Letter to Future Generation intervention (n = 391), participants were asked to think about a child or teenager they knew well, and to write a letter to that child/teenager that the child/teenager would read as an adult in the future (adapted from a prior study (6)). The primary theme for this intervention was Future Thinking, but this intervention is also included under the Self- and Social-Relevance theme because it relates climate change to a socially-close person (Figure 1, main text).

Specifically, participants were first asked to provide the name of a child/teenager they knew well. If they could not think of anyone, they were asked to provide a fictional name. Then participants were asked how well they knew the child/teenager, and how emotionally close they felt to the child/teenager (0 = *Not at all*, 100 = *Extremely*; *N/A* option provided for participants who were imagining a fictional person). Next participants were asked what their relation was to the child/teenager (their own child, their own grandchild, their own niece/nephew, other family relative, a non-family person, *N/A* option provided for participants who were imagining a fictional person). Afterward, participants thought about the kind of person this child/teenager is, and wrote 1-2 sentences each about the child/teenager’s best qualities, passions and hobbies, and likely future accomplishments.

Finally, participants were asked to write a letter to the child/teenager, as if the child/teenager would receive and read the letter as an adult. Specifically, participants were told to imagine that the child/teenager is now a 30-year-old adult in approximately the year 2050 and finds this letter. Participants were asked to tell the child/teenager about “*all of the things you have done and want to do in the future to ensure that they will inherit a healthy, habitable planet. You tell [insert name] about your own personal efforts—however small or large—to confront the complex environmental problems of your time…*”. Participants were also asked to tell the child/teenager about “*how you want to be remembered by them and future generations as someone who did their best to ensure a safe, flourishing world.*” Participants were encouraged to write at least 5-6 sentences, to avoid including identifiable information, and to be sure to write the letter themselves (without the aid of AI, ChatGPT, etc).

Self- & Social Relevance Theme

The following interventions aimed to increase the perceived self-relevance and/or social-relevance of climate change, such as by prompting participants to write comments reflecting on self- and social-relevance, providing information about descriptive social norms, or relating climate change to one’s personal moral values.

News Comments. In the News Comments interventions, participants were encouraged to identify why news about climate change was relevant to themselves or to people in their social networks, using an intervention adapted from prior studies (7). Participants viewed news headlines and ledes about climate change from *The New York Times* and wrote a brief comment about each article. In the *Self-Relevance* condition (n = 396), participants wrote about why each news article was relevant to

themselves, whereas in the *Social-Relevance* condition (n = 392), participants wrote about why it was relevant to people they know. Participants viewed five articles randomly selected from a pool of 26 articles (described in *News Headlines Task)*.

Social Norm Information*.* In the Norm Information interventions, participants received information about normative American attitudes toward climate change and related behaviors. Statistics presented to participants were obtained from national polls of American adults (aged 18+ years) conducted in 2022 or 2023 by the Pew Research Center (8, 9), OnePoll (10), Ipsos (11), Directions Research (12), and the Yale Program on Climate Change Communication (13). Participants viewed 16 statements, randomly selected from a larger set of 24 statements. These statements described the % of Americans who endorsed various attitudes related to climate change (e.g., policy support, willingness to engage in climate action, belief in anthropogenic climate change).

In the *Norm Text* condition (n = 428), participants were informed that they would view statistics about attitudes related to climate change, sourced from recent national polls of American adults. On each trial, they viewed one statement (e.g., "69% of Americans agree that the U.S. should prioritize developing renewable energy sources, like wind and solar."). Below this statement, participants used a 7-point Likert-style scale to rate whether this statistic was lower or higher than expected (1 = *Much lower*, 7 = *Much higher*).

In the *Norm Quiz* condition (n = 426), participants were instead informed that they would complete an interactive quiz in which they would try to guess the missing statistics before the correct answers were revealed. In the Quiz condition, each statement was split into two pages of the survey. On the first page for each statement, participants viewed a version of the statement with the critical statistic omitted (e.g., "__% of Americans agree that the U.S. should prioritize developing renewable energy sources, like wind and solar."). Participants attempted to guess the statistic by using a sliding scale that ranged from 0% to 100%. On the following page, the true value was revealed; participants viewed their guess, the correct answer, and the full intact statement as shown in the Text condition. As in the Text condition, participants also used a 7-point Likert-style scale to rate whether the statistic was lower or higher than expected.

Moral Values. In the Moral Values intervention (n = 420), participants were asked to reflect on how one of their most important moral values was related to climate change. Specifically, participants were told that research in psychology had identified several fundamental moral values that people hold. Participants then read brief descriptions of six moral values adapted from Moral Foundations Theory (14): 1) Care and compassion, 2) Equality of opportunities and outcomes, 3) Proportionality and merit, 4) Loyalty to one's group, 5) Respect for authority and tradition, and 6) Purity/sanctity. Participants selected the moral value that was most important to them, rated how much they identified with that value (0 = *Not at all,* 100 = *Extremely*), and rated how much they thought that value was related to climate change (0 = *Not at all related,* 100 = *Completely related*).

Next, participants were asked to write a few sentences about how their chosen moral value shapes the way they think about and address climate change. Afterward, participants read a persuasive message that further described how their chosen moral value was related to climate change. The message emphasized how human activities were contributing to rising temperatures around the globe, the risks of Earth's natural systems becoming imbalanced, and how the specific moral value was directly related to addressing climate change (e.g., having care and compassion for vulnerable populations who are disproportionately affected by climate change). The message was accompanied by a photo that represented that moral value in the context of the natural environment. Then, participants rated whether the message was convincing, whether they agreed with it, whether most people they care about and spend time with would agree with it, and whether organizations and groups they identify with would support such a message (all items on scale of 1 = *Strongly disagree*, 7 = *Strongly agree*). Finally, participants re-rated how much they thought their chosen moral value was related to climate change (0 = *Not at all related,* 100 = *Completely related*).

Action Impact Theme

The following interventions emphasized the beneficial impacts of engaging in pro-environmental behaviors, including benefits for the environment and for oneself.

Impact Information. In the Impact Information interventions, participants received information about the estimated annual impact (in terms of mitigating greenhouse gas emissions) of actions that individuals could take to help mitigate climate change. Participants viewed information about eight actions, including the six higher-impact individual actions that were also assessed in our outcome measures (see *Climate Action* task). We also included two lower-impact actions in the stimulus set: putting recyclable waste in the recycling bin and setting the thermostat 4° F lower in the winter. Impact estimates were described in terms of pounds of CO_2e (carbon dioxide equivalent) saved, as well as the equivalent number of trees planted. Impact estimates were obtained from a prior meta-analysis assessing the environmental impact of various actions that individuals could take to reduce emissions (15).

Participants first viewed general introductory information about greenhouse gas emissions, climate impact, and individual action. We then provided two example items to demonstrate that some actions are more impactful than others: 1) "Buying 30% of your clothing secondhand would save 110 pounds of CO2 every year, as much as planting 2 trees", and 2) "Reducing your food waste by 70% would save 495 pounds of CO2 every year, as much as planting 10 trees". These two example items remained available for reference throughout the subsequent task, presented at the top of the screen in small text.

In the *Impact Text* condition (n = 418), participants were informed that they would view information about the annual impact of actions that individuals could take to mitigate climate change. On each trial, they viewed one action and the estimated impact (e.g., "Eating a vegan diet would save as much carbon as planting 41 trees (2,006 lb of CO2)"). Below this statement, participants used a 7-point Likert-style scale to rate whether this statistic was lower or higher than expected (1 = *Much lower*, 7 = *Much higher*).

In the *Impact Quiz* condition (n = 416), participants were instead informed that they would complete an interactive quiz game in which they would try to guess the annual impact of these actions. In the Quiz condition, information about each action was split into two pages of the survey. On the first page for each action, participants viewed a version of the statement with the critical statistic omitted (e.g., "Eating a vegan diet would save as much carbon as planting __ trees."). Participants attempted to guess the number of trees by typing a number in a textbox. On the following page, the true value was revealed; participants viewed their guess, the correct answer, and the full intact statement as shown in the Text condition. As in the Text condition, participants also used a 7-point Likert-style scale to rate whether the statistic was lower or higher than expected (1 = *Much lower*, 7 = *Much higher*).

Carbon Footprints. In the Carbon Footprint interventions, participants received information about how various individual actions would reduce one's carbon footprint (i.e., the greenhouse gas emissions associated with one's lifestyle). The primary theme for these interventions was Action Impact, but the Personalized variant of this intervention (described below) was also included in the Self- and Social-Relevance theme (Figure 1).

We first provided general information about carbon footprints: "Your carbon footprint is a measure of the greenhouse gas emissions associated with your lifestyle. Greenhouse gas emissions contribute to climate change (global warming). You can help fight climate change by reducing your carbon footprint. Most of your carbon footprint (about 75%) depends on how you travel, the food you eat, and the energy you use at home. Today, we'll give you some feedback about these three areas." Feedback provided in the intervention focused on 4 individual actions: driving less, flying less, eating less meat, and paying for green energy to power one's home.

In the *Personalized Carbon Footprint* condition (n = 413), participants completed a detailed lifestyle survey. First, participants reported whether they regularly drive a vehicle (at least once per month); if so, they then identified the exact make, model, year, and fuel source of their vehicle. Participants then reported their average driving habits by estimating how many miles they drive for work-related and personal travel on each day of a typical week. Participants were also asked to estimate how many miles they typically drive on long-distance road trips each year, if any. Vehicle emissions data were

obtained from *FuelEconomy.gov,* which provides a comprehensive database of estimated tailpipe CO2 in grams per mile for vehicle models from 1984 through 2024 (16).

Second, participants reported the number of airplane flights taken in a typical year, counting all flights separately (including connections and round trips). Participants reported flights across six duration categories, ranging from very short flights (under 2 hours, regional) to extremely long duration flights (>15 hours, like flying from the central U.S. to Australia). Emissions data for flights were obtained from an interactive online tool provided by *The Guardian* (17).

Third, participants responded to a single item question about their typical diet, self-identifying as an omnivore (eat a variety of animal and meat products), pollotarian (eat poultry and seafood, but no red meat), pescetarian (eat seafood but not meat), flexitarian (eat vegetarian about 70% of the time), vegetarian (eat dairy and eggs, but no meat or seafood), or vegan (eat an entirely plant-based diet). The carbon savings associated with each of these diets (relative to a standard omnivorous diet) were obtained from a prior meta-analysis assessing the environmental impact of various actions that individuals could take (15).

Fourth, participants estimated their home energy usage by reporting their current U.S. state of residence and identifying energy sources used to power or heat their home (electricity, natural gas, fuel oil, and/or propane). Participants then estimated the monthly cost associated with each of the energy sources (e.g., typical monthly electricity bills). If participants did not know the energy sources used to power their home or were unable to estimate cost for these energy sources, they were permitted to skip this estimation step and view information about an average household in their state instead of their personalized household footprint. To estimate energy/fuel consumption from monthly cost, we used formulas from the Carbon Footprint calculator provided by the U.S. Environmental Protection Agency (EPA) (18). To estimate the emissions associated with electricity use (which varies depending on the fuel mix used to power a regional electrical grid), we used EPA data detailing emissions per kWh in each U.S. state (19). For participants who opted to view results for an average household in their state, we instead provided estimates from a U.S. Energy Information Administration (EIA) database (20).

Finally, participants received personalized feedback about the carbon emissions associated with their current driving, flying, diet, and home energy. For each of these categories, participants viewed estimates about how much carbon would be saved by making various lifestyle changes (reducing driving or flying by 50%, switching to various alternative diets, or paying a monthly fee to obtain 50% of your home energy from renewable sources), as well as suggested strategies for implementing these lifestyle changes (e.g., taking public transportation, choosing closer travel destinations, or replacing beef with chicken). Feedback about each action category (driving, flying, diet, and energy) was provided on separate pages with descriptive headings and illustrations.

In the *General Carbon Footprint* condition (n = 428), participants viewed the same feedback pages, but with carbon footprints and savings calculated for an average U.S. resident. Estimated annual emissions from a typical passenger vehicle were obtained from the EPA (21). Average per-capita emissions from domestic flights were obtained from a prior report (22). As in the Personalized condition, emissions associated with various diets were obtained from a prior meta-analysis (15). Average U.S. household energy consumption statistics were obtained from the EIA database described above (20).

Personal Benefits. In the Personal Benefits intervention (n = 370), participants were asked to brainstorm *personal* benefits that could arise from engaging in pro-environmental behaviors, with an emphasis on short-term benefits. The primary theme for this intervention was Action Impact, but this intervention was also included under the Self- and Social-Relevance and Future Thinking themes; the task involved thinking about the near future personal impact of various actions (Figure 1, main text).

Participants were informed that they would view a series of ten actions that can benefit the environment as well as themselves and close others. For each action, participants were instructed to brainstorm personal benefits that they could experience as a result of engaging in the action regularly over the next six months. Participants were prompted to be *creative* (generating many benefits pertaining to different domains, such as health, well-being, relationships, and finances) and *specific* (breaking down broad benefits like “being healthier” into specific details like “better cardio fitness” and “lower stress”).

Participants who already did an action regularly were instructed to describe the benefits they currently experience, as opposed to listing anticipated future benefits.

For each action, participants typed into a textbox to describe a benefit. Participants could click a button to generate additional textboxes to report up to 20 benefits for a given action. Providing at least one benefit was required to advance. Participants repeated this process for a total of ten actions, presented in a randomized order: 1) eating less beef or lamb, 2) eating more vegetarian and/or vegan meals, 3) driving a fuel-powered vehicle less frequently (e.g., by walking, biking, taking public transit, or carpooling), 4) flying by airplane less frequently (e.g., by taking the train, choosing closer destinations, or traveling less), 5) contacting local or national representatives to ask them to address climate change, 6) signing petitions for local or national policies that aim to address climate change, 7) volunteering for organizations or political candidates that aim to address climate change, 8) donating money to organizations or political candidates that aim to address climate change, 9) paying for renewable energy to power one's home, and 10) talking to family, friends, or colleagues about climate change.

Outcome Measures

After completing an intervention task (or after consent in the no-intervention Control group), participants completed the Climate Action, News Headlines, and Petitions Tasks (described below) in a randomized order. In the News Comments interventions, however, participants always completed the News Headlines task first, because these interventions modified this task by adding a writing component. After the primary tasks, participants completed a series of secondary measures in a randomized order.

Climate Action Task. Participants were asked about 12 actions that could have positive or negative effects on climate change. The list of actions included seven individual actions (eating beef or lamb, eating vegetarian meals, eating vegan meals, driving a gas- or diesel-powered vehicle, flying by airplane, recycling, and paying for renewable energy to power one's home) and five collective actions (donating, volunteering, signing petitions, contacting representatives, and talking to others about climate change). In a pilot study, we assessed beliefs about various pro-environmental behaviors, identifying actions that were feasible but not yet widely adopted (i.e., few people already engage in the action as much as possible). From this list of actions, we selected a subset of target actions that were recommended by climate scientists and associated with greater reduction of greenhouse gas emissions (23).

Actions were presented in a randomized order, with a single action presented per page. For each action, participants first reported their current frequency of engaging in the action. To describe current air travel habits, participants reported the number of flights taken in the past year, across six duration categories ranging from very short flights (under 2 hours) to extremely long flights (greater than 15 hours). To describe current donation behavior, participants input a number to approximate the total sum (in USD) that they donated in the past year to support organizations or candidates that aim to address climate change. To describe current payments for renewable energy, participants input a number to approximate the amount (in USD), if any, that they currently pay to their electricity provider to power their home with renewable energy. For dietary actions, recycling, and driving, participants reported current frequency on a 9-point scale (1 = *Never*, 9 = *Multiple times per day*). For collective actions (with the exception of donations, described above), participants used a similar 9-point frequency scale (1 = *Never / Almost never*, 9 = *Every day*). These estimates of current frequency were z-scored within-item to account for discrepancies in scale, then included in statistical models as a covariate.

News Headlines Task. Participants viewed a set of five news headlines about climate change (consisting of a title and an accompanying lede), randomly selected from a larger set of 26 headlines sourced from the New York Times. For each article presented, participants used a scale from 0 (*strongly disagree*) to 100 (*strongly agree*), to rate their intentions to share the article broadly on social media ("broadcast" sharing) or directly with someone they know ("narrowcast" sharing). Using the same rating scale, participants also rated their desire to read the article and perceived self-relevance and social-relevance of the information.

Secondary outcome measures

In addition to the primary outcome measures of interest, we included a number of secondary outcomes. These included perceived self- and social-relevance of news headlines and petitions, self-efficacy, emotions about climate change, psychological distance, perceived risk and concern about climate change. We also collected several individual differences measures: self-reported knowledge about climate change, uncertainty and skepticism about climate change, climate change anxiety, personal experience with climate change, and the Index of Autonomous Functioning scale. Lastly, some interventions included additional measures that were intended to be included as covariates in additional analyses beyond the scope of this report (e.g., vividness and affect ratings collected after Guided Imagination exercises). Measures that were included across conditions are briefly described below.

We measured climate change **self-efficacy** with a subset of four items selected from the Climate Change Attitude Survey (24). Participants responded with a 5-point agreement scale (1 = *Strongly disagree*, 5 = *Strongly agree*). Similarly, we used 5-point agreement scales (1 = *Strongly disagree*, 5 = *Strongly agree*) to measure the following **emotions about climate change**: anxiety, hope, hopelessness, determination, disengagement, uncertainty, anger, and sadness**.** The **psychological distance** of climate change was measured using separate subscales for spatial, temporal, and social distance (25). Spatial and social distance were assessed with two items per subscale, measured using a 5-point agreement scale. Temporal distance was measured with a single item, using a 7-point scale, about when humanity would experience widespread harmful effects due to climate change (1 = *We are already feeling the effects* … 7 = *Never*). **Perceived risk and concern** were measured using 5-point agreement rating across four items. Five-point agreement scales were used to measure **knowledge** (2 items), and **uncertainty/skepticism** (4 items) (25).

In the Control group, we administered several additional scales. These measures were included to equate the total task time across intervention and Control groups, and to collect pilot data for other future studies. To measure propensity for **autonomous expression**, we included the Index of Autonomous Functioning (26). We also measured **climate change anxiety** (27); within this scale, we separated subscales for items about broader climate anxiety and items about **personal experience with climate change**. These measures were not analyzed for the present study.

Statistical Analysis

Bayesian analyses were conducted with the *brms* package (version 2.21.0) (28). We compared point estimates for each intervention group with the control group using the *emmeans* package (version 1.10.3) (29) and visualized results with *ggplot2* (version 3.5.1) (30). Other packages used in data processing included dplyr (31), tidyr (32), and stringr (33).

For outcome measures with more than one observation per participant (measures from the Climate Action task, News Headlines task, and Petitions task), we used Bayesian linear mixed-effects regression models. Binary outcomes (e.g., self-reported petition signing) were analyzed with Bayesian generalized linear mixed-effects models. Random effects varied across models; detailed information about each model is provided in the caption for the corresponding table of parameter estimates. In all mixed-effects models, we aimed to identify a random effects structure that was supported by the experimental design, striking a balance between generalizability, Type-I error rate, power, and model fit (34, 35). We began by including random intercepts for participants and stimuli, as well as random slopes for all variables of interest that varied within-participant (if applicable). We then simplified the random effects structure as needed, first removing elements that explained the least variance, to achieve convergence without overfitting. Models used for Climate Action Task measures (action intentions and perceived impact) included random intercepts for participants. Models used to assess measures from the News Headlines and Petitions task (sharing intentions) included random intercepts for participants and stimuli (i.e., each news headline or petition). For outcome measures with only a single observation or composite score for each participant, we used Bayesian linear regression models.

We also preprocessed data to account for extreme observations in the Climate Action Task. Individual open-ended numeric responses for measures of current behaviors (i.e., amount of money donated or paid for renewable energy and the number of flights taken) that were implausibly high were

winsorized to the 99th percentile (13 observations for donations, 13 observations for energy, and 68 observations for flights). For analyses of action intentions, we also excluded trials in cases where it was not possible for the participant to engage in the action more in the future (i.e., current behavior was reported at the maximum possible level). For example, an individual who does not own a car and never drives cannot reduce driving further; an individual who always eats a vegan diet cannot reduce meat consumption further. However, we also conducted the same analyses with these "maxed-out" actions included and obtained results that were consistent with the results reported in the main text (Table S16).

Supplementary Results

Perceived Self- and Social-Relevance of News Headlines

During the News Headlines task, all participants rated the extent to which they perceived that a given news headline was relevant to themself or relevant to people they know. In separate models, we compared self- and social-relevance ratings across conditions (Table S10). As expected, the two variants of the News Comments intervention (in which participants wrote comments about why these news headlines were relevant to themselves or others) had the strongest effects, substantially increasing perceived self-relevance and social-relevance relative to the Control group. The Letter to Future Generation intervention also moderately increased perceived self- and social-relevance. Interestingly, the Quiz condition within the Social Norms intervention had a backfire effect, decreasing both self- and social-relevance relative to the Control group.

Overall, these findings are consistent with prior evidence that perceived self- and social-relevance is a key mechanism that accounts for intentions to share information (7). The two News Comments interventions and the Letter to Future Generation intervention, the most effective interventions for motivating individuals to share news articles and petitions about climate change, all increased the perceived self-relevance and social-relevance of information about climate change. Furthermore, these findings suggest that relating climate change to specific close others can increase perceived self- and social-relevance of climate change information, but learning about general normative attitudes may have the opposite effect.

Psychological Distance of Climate Change

Next, we investigated psychological distance associated with climate change (i.e., how remote the effects of climate change feel). We were interested in whether the interventions would decrease the psychological distance of climate change. The psychological distance measure included three subscales: temporal distance (i.e., when we will see widespread effects of climate change), geographic distance (i.e., whether climate change will impact your local area), and social distance (i.e., whether climate change will impact you and people like you). We examined each of these subscales separately, using Bayesian linear regression to compare each intervention condition with the Control group.

Results for the three psychological distance subscales are reported in Table S11. Only the Personal Benefits decreased perceived temporal distance relative to the Control group. None of the interventions influenced geographic distance. The Social Norms (Quiz) and Moral Values interventions, both within the Relevance theme, decreased social distance relative to the Control group. Overall, we saw limited effects on psychological distance, suggesting that the benefits of the leading interventions were not driven by reducing the perceived distance of climate change.

Self-Efficacy Related to Climate Change

We calculated composite scores from a subset of four items related to self-efficacy selected from the Climate Change Attitude Survey (91). These survey items assessed belief in our ability (as individuals and as a society) to take action to mitigate climate change. Using Bayesian linear regression, we

compared self-efficacy scores in each intervention group with the Control group. In descending order of effectiveness, the Personal Benefits, Moral Values, Letter to Future Generation, Guided Imagination (Promotion-Other), Impact Information (Quiz), Action Planning (Collective), and News Comments (Social-Relevance) interventions all increased self-efficacy relative to the Control group (Table S12).

Perceived Risk of Climate Change

To assess perceived risk, we calculated composite scores from a four-item scale measuring concern and perceived risk related to climate change (see SI Appendix). The two prevention-focused variants of the Guided Imagination intervention (Prevention-Self and Prevention-Other) both increased concern and perceived risk relative to the Control group (Table S13). No interventions decreased concern and perceived risk.

Emotions Related to Climate Change

All estimates are reported in Table S14. The Prevention-Self and Prevention-Other variants of the Guided Imagination intervention both increased anger relative to the Control group, as did the Letter to Future Generation intervention. No interventions decreased anger relative to the Control group. The Prevention-Self variant of the Guided Imagination intervention slightly increased anxiety ratings relative to the Control group. The Moral Values and Carbon Footprint (Personalized) interventions decreased anxiety relative to the Control group. The Prevention-Self variant of the Guided Imagination intervention increased sadness relative to the Control group; no interventions decreased sadness. The Letter to Future Generation, Guided Imagination (Promotion-Self), and Moral Values interventions increased hope relative to the Control group; no interventions decreased hope. The Guided Imagination (Promotion-Self) intervention also decreased hopelessness; no interventions increased hopelessness. The Carbon Footprint (Personalized) intervention decreased uncertainty; no interventions increased uncertainty. The Letter to Future Generation intervention, Action Planning (Individual and Collective variants), Moral Values, Guided Imagination (Promotion variants), and News Comments (Social-Relevance) interventions all increased determination; no interventions decreased determination. The Letter to Future Generation intervention decreased disengagement; no interventions increased disengagement.

Petitions Task: Signing Intentions, Link Clicks and Self-Reported Signing

In the Petitions task, participants viewed three petitions about climate change, adapted from real online petitions. We collected three measures related to petition signing, described below; results are reported in Table S15. Participants first rated their willingness to sign each petition. Using Bayesian linear mixed-effects regression (including random intercepts for participants and stimuli), we compared signing intentions among conditions. The Letter to Future Generation and Personal Benefits interventions increased petition signing intentions relative to the Control group. No interventions decreased signing intentions.

After viewing the abbreviated petition preview and providing ratings, participants were given the option to click a link to view the petition and sign it, if desired. We recorded whether participants clicked on the links. However, due to technical errors with the click-tracking code, clicks were not recorded for a subset of participants, and clicks that were recorded were not labeled with the corresponding petitions. Therefore, for this outcome measure, we used Bayesian linear regression to compare the total number of links clicked (up to 3 clicks) among conditions. This analysis is exploratory and underpowered due to the technical issues that led to data loss. The Moral Values, Letter, and Personal Benefits conditions all increased clicks on petition links relative to the Control group. No interventions decreased clicks.

Lastly, participants self-reported whether or not they had actually signed each petition. Using Bayesian generalized linear mixed-effects regression (including random intercepts for participants and stimuli), we compared signing outcomes (0=did not sign, 1=signed) among intervention arms. None of the intervention groups differed from the Control group.

Table S1. Descriptive statistics of demographic variables in samples 1 and 2.

**Demographic**	**Sample 1**	**Sample 2**
**Age** – *Mean (SD)*	M = 39.3, SD = 13.7, Range [18, 88]	M = 40.6, SD = 13.6, Range [18, 86]
**Gender** – *N (%)*		
Woman	3336 (51.8%)	591 (50.0%)
Man	2892 (44.9%)	557 (47.2%)
Nonbinary, genderqueer, agender, gender fluid, or other gender identity	193 (3.0%)	29 (2.5%)
Preferred not to answer	22 (0.3%)	4 (0.3%)
**Hispanic/Latinx** – *N (%)*		
Yes	673 (10.4%)	103 (8.7%)
No	5744 (89.2%)	1072 (90.8%)
Preferred not to answer	26 (0.4%)	5 (0.4%)
**Race** – *N (%)*		
White	4373 (67.8%)	751 (63.6%)
Black or African American	884 (13.7%)	219 (18.5%)
East Asian	289 (4.5%)	48 (4.1%)
Southeast Asian	167 (2.6%)	25 (2.1%)
South Asian	114 (1.8%)	24 (2.0%)
American Indian or Alaskan Native	34 (0.5%)	6 (0.5%)
Native Hawaiian or Other Pacific Islander	9 (0.1%)	2 (0.1%)
Two or more races	386 (6.0%)	67 (5.7%)
Preferred not to answer	60 (0.9%)	8 (0.7%)
Other racial identity not listed	127 (2.0%)	31 (2.6%)

Table S2. Descriptive statistics (mean and standard deviation) for all primary outcome measures for the no-intervention Control group.

DV	Mean	SD
Action Intentions (1-7 scale)	4.39	1.05
Perceived Impact (1-7 scale)	3.64	1.55
Sharing Articles – Broadcast (1-100 scale)	27.72	32.31
Sharing Articles – Narrowcast (1-100 scale)	33.51	33.55
Sharing Petitions – Broadcast (1-100 scale)	29.57	34.93
Sharing Petitions – Narrowcast (1-100 scale)	35.44	35.88

Table S3. Estimated treatment effects (Intervention – Control, comparing medians of posterior distributions) for action intentions, across all action categories. Refer to Table S3 for effects subset by action category. Point estimates are reported next to 95% credible intervals (denoted in brackets).

**Contrast (intervention > control)**	**Estimate [95% CI]**
News Comments (Self-Rel)	0.009 [-0.06, 0.08]
News Comments (Social-Rel)	0.054 [-0.01, 0.12]
Social Norms (Text)	-0.003 [-0.07, 0.06]
Social Norms (Quiz)	0.022 [-0.04, 0.08]
Moral Values	0.051 [-0.02, 0.11]
Imagination (Prevention-Self)	0.141 [0.08, 0.21]
Imagination (Prevention-Other)	0.077 [0.01, 0.14]
Imagination (Promotion-Self)	0.064 [0, 0.13]
Imagination (Promotion-Other)	0.042 [-0.03, 0.11]
Action Planning (Individual)	0.103 [0.04, 0.17]
Action Planning (Collective)	0.077 [0.01, 0.14]
Letter to Future Gen	0.133 [0.06, 0.2]
Impact Information (Text)	0.046 [-0.02, 0.11]
Impact Information (Quiz)	0.054 [-0.01, 0.12]
Carbon Footprint (General)	0.028 [-0.04, 0.09]
Carbon Footprint (Personalized)	-0.008 [-0.07, 0.06]
Personal Benefits	0.095 [0.03, 0.16]

Table S4. Estimated treatment effects (Intervention – Control, comparing medians of posterior distributions) for action intentions, subset by action category (collective actions, conversations about climate change, dietary changes, paying for green energy at home, recycling, and transit-related actions).

Contrast (intervention > control)	Collective Actions (Estimate [95% CI)	Conversations (Estimate [95% CI)	Dietary Actions (Estimate [95% CI)
News Comments (Self-Rel)	0.003 [-0.07, 0.08]	0.09 [-0.02, 0.21]	-0.03 [-0.11, 0.05]
News Comments (Social-Rel)	0.073 [0, 0.15]	0.068 [-0.05, 0.18]	-0.001 [-0.09, 0.08]
Social Norms (Text)	-0.018 [-0.09, 0.06]	0.043 [-0.07, 0.16]	0.039 [-0.05, 0.12]
Social Norms (Quiz)	0.023 [-0.05, 0.1]	0.011 [-0.1, 0.12]	0.024 [-0.06, 0.11]
Moral Values	0.069 [-0.01, 0.14]	0.072 [-0.04, 0.19]	0.036 [-0.05, 0.12]
Imagination (Prevention-Self)	0.181 [0.11, 0.27]	0.114 [0, 0.23]	0.092 [0.01, 0.17]
Imagination (Prevention-Other)	0.054 [-0.02, 0.13]	0.084 [-0.03, 0.2]	0.091 [0, 0.17]
Imagination (Promotion-Self)	0.095 [0.02, 0.18]	0.084 [-0.03, 0.2]	0.034 [-0.06, 0.12]
Imagination (Promotion-Other)	0.055 [-0.03, 0.13]	0.015 [-0.1, 0.13]	-0.032 [-0.12, 0.06]
Action Planning (Individual)	0.032 [-0.04, 0.11]	0.057 [-0.06, 0.17]	0.221 [0.13, 0.3]
Action Planning (Collective)	0.161 [0.09, 0.24]	0.249 [0.14, 0.37]	-0.039 [-0.13, 0.04]
Letter to Future Gen	0.177 [0.1, 0.25]	0.221 [0.1, 0.34]	0.058 [-0.02, 0.14]
Impact Information (Text)	0.048 [-0.02, 0.12]	0.011 [-0.1, 0.12]	0.097 [0.01, 0.18]
Impact Information (Quiz)	0.062 [-0.01, 0.14]	0.129 [0.02, 0.24]	0.083 [0.01, 0.17]
Carbon Footprint (General)	0.003 [-0.07, 0.08]	0.047 [-0.06, 0.16]	0.046 [-0.04, 0.13]
Carbon Footprint (Personalized)	-0.048 [-0.13, 0.03]	-0.052 [-0.17, 0.06]	0.056 [-0.03, 0.14]
Personal Benefits	0.086 [0.01, 0.16]	0.148 [0.03, 0.26]	0.118 [0.03, 0.2]
**Contrast (intervention > control)**	**Renewable Energy** (Estimate [95% CI)	**Recycling** (Estimate [95% CI)	**Transit Actions** (Estimate [95% CI)
News Comments (Self-Rel)	0.071 [-0.04, 0.19]	-0.008 [-0.16, 0.14]	0.015 [-0.08, 0.11]
News Comments (Social-Rel)	0.039 [-0.08, 0.15]	0.092 [-0.05, 0.25]	0.069 [-0.03, 0.17]
Social Norms (Text)	-0.033 [-0.15, 0.08]	0.02 [-0.13, 0.16]	-0.079 [-0.18, 0.02]
Social Norms (Quiz)	0.007 [-0.1, 0.12]	-0.047 [-0.2, 0.09]	0.037 [-0.07, 0.13]

Moral Values	-0.027 [-0.14, 0.08]	0.03 [-0.12, 0.17]	0.062 [-0.03, 0.16]
Imagination (Prevention-Self)	0.105 [-0.01, 0.22]	0.068 [-0.09, 0.21]	0.176 [0.07, 0.28]
Imagination (Prevention-Other)	0.068 [-0.05, 0.19]	0.014 [-0.14, 0.17]	0.121 [0.02, 0.22]
Imagination (Promotion-Self)	0.057 [-0.06, 0.17]	0.075 [-0.08, 0.22]	0.002 [-0.1, 0.11]
Imagination (Promotion-Other)	0.046 [-0.07, 0.16]	0.121 [-0.03, 0.27]	0.132 [0.03, 0.23]
Action Planning (Individual)	0.111 [0, 0.23]	0.083 [-0.07, 0.23]	0.109 [0.01, 0.21]
Action Planning (Collective)	-0.086 [-0.2, 0.03]	-0.005 [-0.16, 0.14]	0.042 [-0.06, 0.15]
Letter to Future Gen	0.161 [0.05, 0.28]	-0.004 [-0.16, 0.15]	0.084 [-0.02, 0.18]
Impact Information (Text)	0.054 [-0.06, 0.17]	0.038 [-0.1, 0.18]	-0.073 [-0.17, 0.03]
Impact Information (Quiz)	-0.029 [-0.14, 0.09]	-0.017 [-0.17, 0.12]	-0.017 [-0.11, 0.08]
Carbon Footprint (General)	-0.044 [-0.16, 0.07]	0.008 [-0.13, 0.16]	0.072 [-0.03, 0.17]
Carbon Footprint (Personalized)	-0.093 [-0.21, 0.02]	-0.011 [-0.16, 0.13]	0.04 [-0.06, 0.14]
Personal Benefits	0.037 [-0.08, 0.16]	0.109 [-0.05, 0.26]	0.069 [-0.03, 0.17]

Table S5. Estimated treatment effects (Intervention – Control, comparing medians of posterior distributions) for perceived impact of pro-environmental behaviors, across all action categories. Refer to Table S5 for effects subset by action category.

**Contrast (intervention > control)**	**Estimate [95% CI]**
News Comments (Self-Rel)	0.072 [-0.02, 0.16]
News Comments (Social-Rel)	0.163 [0.08, 0.25]
Social Norms (Text)	0.045 [-0.04, 0.13]
Social Norms (Quiz)	0.098 [0.02, 0.18]
Moral Values	0.297 [0.21, 0.38]
Imagination (Prevention-Self)	0.074 [-0.02, 0.16]
Imagination (Prevention-Other)	0.072 [-0.02, 0.16]
Imagination (Promotion-Self)	0.135 [0.05, 0.23]
Imagination (Promotion-Other)	0.134 [0.04, 0.22]
Action Planning (Individual)	0.107 [0.02, 0.19]
Action Planning (Collective)	0.238 [0.14, 0.32]
Letter to Future Gen	0.379 [0.29, 0.47]
Impact Information (Text)	0.195 [0.11, 0.28]
Impact Information (Quiz)	0.292 [0.21, 0.37]
Carbon Footprint (General)	0.177 [0.09, 0.26]
Carbon Footprint (Personalized)	0.156 [0.07, 0.24]
Personal Benefits	0.358 [0.27, 0.44]

Table S6. Estimated treatment effects (Intervention – Control, comparing medians of posterior distributions) for perceived impact of pro-environmental behaviors, subset by action category (collective actions, conversations about climate change, dietary changes, paying for green energy at home, recycling, and transit-related actions).

**Contrast (intervention > control)**	**Collective** Estimate [95% CI]	**Conversations** Estimate [95% CI]	**Diet** Estimate [95% CI]
News Comments (Self-Rel)	0.08 [-0.01, 0.17]	0.115 [0, 0.23]	0.077 [-0.02, 0.17]
News Comments (Social-Rel)	0.149 [0.06, 0.24]	0.134 [0.01, 0.24]	0.189 [0.1, 0.29]
Social Norms (Text)	0.017 [-0.07, 0.1]	0.058 [-0.05, 0.16]	0.076 [-0.01, 0.16]
Social Norms (Quiz)	0.075 [-0.01, 0.17]	0.04 [-0.07, 0.15]	0.155 [0.07, 0.25]
Moral Values	0.332 [0.24, 0.42]	0.405 [0.3, 0.52]	0.273 [0.18, 0.37]
Imagination (Prevention-Self)	0.105 [0.02, 0.2]	0.154 [0.04, 0.26]	0.07 [-0.03, 0.16]
Imagination (Prevention-Other)	0.075 [-0.01, 0.17]	0.113 [0, 0.23]	0.116 [0.02, 0.21]
Imagination (Promotion-Self)	0.123 [0.03, 0.22]	0.19 [0.08, 0.31]	0.167 [0.07, 0.26]
Imagination (Promotion-Other)	0.161 [0.07, 0.25]	0.145 [0.03, 0.26]	0.032 [-0.06, 0.13]
Action Planning (Individual)	0.081 [-0.01, 0.17]	0.129 [0.02, 0.24]	0.102 [0.01, 0.2]
Action Planning (Collective)	0.271 [0.18, 0.36]	0.335 [0.21, 0.45]	0.217 [0.12, 0.31]
Letter to Future Gen	0.447 [0.35, 0.53]	0.491 [0.37, 0.6]	0.364 [0.27, 0.46]
Impact Information (Text)	0.104 [0.02, 0.2]	0.057 [-0.06, 0.17]	0.348 [0.25, 0.44]
Impact Information (Quiz)	0.171 [0.09, 0.26]	0.274 [0.16, 0.39]	0.411 [0.32, 0.5]
Carbon Footprint (General)	0.088 [0, 0.18]	0.131 [0.02, 0.24]	0.283 [0.19, 0.37]
Carbon Footprint (Personalized)	0.09 [0.01, 0.18]	0.07 [-0.04, 0.18]	0.282 [0.19, 0.37]
Personal Benefits	0.392 [0.3, 0.48]	0.422 [0.31, 0.54]	0.329 [0.23, 0.42]

Table S6 (continued).

Contrast (intervention > control)	Energy<br>Estimate [95% CI]	Recycling<br>Estimate [95% CI]	Transit<br>Estimate [95% CI]
News Comments (Self-Rel)	0.08 [-0.03, 0.2]	0.109 [-0.01, 0.22]	0.009 [-0.09, 0.11]
News Comments (Social-Rel)	0.162 [0.04, 0.27]	0.173 [0.06, 0.28]	0.168 [0.07, 0.27]
Social Norms (Text)	0.053 [-0.06, 0.16]	0.063 [-0.04, 0.18]	0.019 [-0.08, 0.11]
Social Norms (Quiz)	0.084 [-0.03, 0.2]	0.093 [-0.02, 0.2]	0.119 [0.02, 0.22]
Moral Values	0.212 [0.1, 0.33]	0.385 [0.28, 0.5]	0.168 [0.07, 0.27]
Imagination (Prevention-Self)	0.094 [-0.02, 0.21]	0.033 [-0.08, 0.15]	0.034 [-0.07, 0.13]
Imagination (Prevention-Other)	0.008 [-0.1, 0.12]	0.049 [-0.07, 0.16]	0.084 [-0.02, 0.18]
Imagination (Promotion-Self)	0.148 [0.04, 0.27]	0.097 [-0.02, 0.21]	0.128 [0.03, 0.23]
Imagination (Promotion-Other)	0.206 [0.1, 0.32]	0.147 [0.03, 0.27]	0.134 [0.03, 0.23]
Action Planning (Individual)	0.122 [0.01, 0.24]	0.136 [0.03, 0.26]	0.153 [0.05, 0.25]
Action Planning (Collective)	0.182 [0.07, 0.3]	0.196 [0.07, 0.3]	0.175 [0.08, 0.28]
Letter to Future Gen	0.359 [0.25, 0.48]	0.422 [0.31, 0.53]	0.172 [0.07, 0.27]
Impact Information (Text)	0.186 [0.08, 0.3]	0.088 [-0.03, 0.2]	0.289 [0.19, 0.38]
Impact Information (Quiz)	0.321 [0.21, 0.43]	0.227 [0.11, 0.33]	0.382 [0.28, 0.48]
Carbon Footprint (General)	0.229 [0.12, 0.33]	0.192 [0.08, 0.3]	0.229 [0.14, 0.33]
Carbon Footprint (Personalized)	0.113 [0.01, 0.23]	0.168 [0.06, 0.28]	0.159 [0.06, 0.26]
Personal Benefits	0.308 [0.2, 0.43]	0.384 [0.27, 0.5]	0.218 [0.12, 0.32]

Table S7. Estimated treatment effects (Intervention – Control, comparing medians of posterior distributions) for intentions to share news articles about climate change, either broadly on social media (“broadcast”) or directly with a known other (“narrowcast”).

	**Broadcast**	**Narrowcast**
**Contrast (intervention > control)**	Estimate [95% CI]	Estimate [95% CI]
News Comments (Self-Rel)	0.419 [0.32, 0.52]	0.33 [0.23, 0.42]
News Comments (Social-Rel)	0.48 [0.38, 0.58]	0.438 [0.34, 0.54]
Social Norms (Text)	0.006 [-0.1, 0.1]	-0.007 [-0.09, 0.09]
Social Norms (Quiz)	-0.037 [-0.13, 0.07]	-0.077 [-0.16, 0.02]
Moral Values	0.151 [0.05, 0.25]	0.174 [0.08, 0.27]
Imagination (Prevention-Self)	0.069 [-0.03, 0.18]	0.089 [-0.01, 0.19]
Imagination (Prevention-Other)	0.039 [-0.07, 0.15]	0.023 [-0.08, 0.13]
Imagination (Promotion-Self)	-0.037 [-0.14, 0.07]	-0.062 [-0.16, 0.04]
Imagination (Promotion-Other)	0.017 [-0.09, 0.12]	0.006 [-0.09, 0.11]
Action Planning (Individual)	0.068 [-0.03, 0.17]	0.003 [-0.09, 0.1]
Action Planning (Collective)	0.111 [0, 0.21]	0.119 [0.01, 0.21]
Letter to Future Gen	0.289 [0.19, 0.39]	0.32 [0.23, 0.42]
Impact Information (Text)	-0.02 [-0.11, 0.08]	-0.058 [-0.15, 0.04]
Impact Information (Quiz)	0.108 [0, 0.21]	0.054 [-0.04, 0.15]
Carbon Footprint (General)	-0.046 [-0.15, 0.05]	-0.045 [-0.14, 0.05]
Carbon Footprint (Personalized)	-0.033 [-0.14, 0.06]	-0.056 [-0.15, 0.04]
Personal Benefits	0.129 [0.03, 0.23]	0.139 [0.05, 0.24]

Table S8. Estimated treatment effects (Intervention – Control, comparing medians of posterior distributions) for intentions to share petitions about climate change, either broadly on social media ("broadcast") or directly with a known other ("narrowcast").

Contrast (intervention > control)	Broadcast<br>Estimate [95% CI]	Narrowcast<br>Estimate [95% CI]
News Comments (Self-Rel)	0.188 [0.07, 0.3]	0.119 [0.01, 0.23]
News Comments (Social-Rel)	0.219 [0.11, 0.33]	0.183 [0.08, 0.29]
Social Norms (Text)	0.018 [-0.1, 0.13]	0 [-0.1, 0.1]
Social Norms (Quiz)	-0.03 [-0.14, 0.08]	-0.047 [-0.15, 0.06]
Moral Values	0.086 [-0.02, 0.19]	0.107 [0, 0.21]
Imagination (Prevention-Self)	0.063 [-0.05, 0.17]	0.073 [-0.04, 0.18]
Imagination (Prevention-Other)	-0.015 [-0.13, 0.1]	-0.025 [-0.13, 0.09]
Imagination (Promotion-Self)	-0.056 [-0.17, 0.06]	-0.093 [-0.2, 0.02]
Imagination (Promotion-Other)	-0.023 [-0.14, 0.09]	-0.054 [-0.17, 0.05]
Action Planning (Individual)	0.023 [-0.09, 0.14]	-0.047 [-0.16, 0.06]
Action Planning (Collective)	0.057 [-0.06, 0.16]	0.073 [-0.04, 0.18]
Letter to Future Gen	0.259 [0.14, 0.37]	0.313 [0.21, 0.42]
Impact Information (Text)	-0.022 [-0.13, 0.09]	-0.038 [-0.14, 0.07]
Impact Information (Quiz)	0.104 [-0.01, 0.21]	0.066 [-0.04, 0.17]
Carbon Footprint (General)	-0.077 [-0.18, 0.04]	-0.092 [-0.2, 0.01]
Carbon Footprint (Personalized)	-0.066 [-0.17, 0.04]	-0.102 [-0.2, 0.01]
Personal Benefits	0.116 [-0.01, 0.23]	0.171 [0.07, 0.28]

Table S9. Summary of all secondary outcome measures. **+** indicates a significant positive intervention effect (greater than Control group), whereas **–** indicates a significant negative intervention effect (less than Control group). Shaded cells identify the intervention with the strongest effect for each outcome measures.

Intervention Condition	Self-Relevance	Social-Relevance	Temporal Distance	Geographic Distance	Social Distance	Perceived Risk	Self-Efficacy	Petition Signing
News Comments (Self-Rel)	+	+						
News Comments (Social-Rel)	+	+					+	
Social Norms (Text)								
Social Norms (Quiz)	–	–			–			
Moral Values					–		+	
Imagination (Prevention-Self)						+		
Imagination (Prevention-Other)						+		
Imagination (Promotion-Self)								
Imagination (Promotion-Other)							+	
Action Planning (Individual)								
Action Planning (Collective)							+	
Letter to Future Gen	+	+					+	+
Impact Information (Text)								
Impact Information (Quiz)							+	
Carbon Footprint (General)								
Carbon Footprint (Personalized)								
Personal Benefits			–				+	+

Table S9 (continued)

Intervention Condition	Anger	Anxiety	Sadness	Hope	Hope-lessness	Deter-mination	Dis-engagement	Uncertainty
News Comments (Self-Rel)								
News Comments (Social-Rel)						+		
Social Norms (Text)								
Social Norms (Quiz)								
Moral Values		–		+		+		
Imagination (Prevention-Self)	+	+	+					
Imagination (Prevention-Other)	+							
Imagination (Promotion-Self)				+	–	+		
Imagination (Promotion-Other)						+		
Action Planning (Individual)						+		
Action Planning (Collective)						+		
Letter to Future Gen	+			+		+	–	
Impact Information (Text)								
Impact Information (Quiz)								
Carbon Footprint (General)								
Carbon Footprint (Personalized)		–						–
Personal Benefits								

Table S10. Estimated treatment effects (Intervention – Control, comparing medians of posterior distributions) for perceived self-relevance and social-relevance of climate-related news headlines.

Contrast (intervention > control)	**Self-Relevance** Estimate [95% CI]	**Social-Relevance** Estimate [95% CI]
News Comments (Self-Rel)	0.366 [0.27, 0.45]	0.31 [0.22, 0.4]
News Comments (Social-Rel)	0.435 [0.35, 0.52]	0.452 [0.37, 0.54]
Social Norms (Text)	-0.031 [-0.12, 0.06]	-0.065 [-0.15, 0.02]
Social Norms (Quiz)	-0.091 [-0.18, 0]	-0.09 [-0.17, 0]
Moral Values	0.091 [0.01, 0.18]	0.027 [-0.06, 0.11]
Imagination (Prevention-Self)	0.092 [0.01, 0.19]	0.069 [-0.02, 0.16]
Imagination (Prevention-Other)	0.023 [-0.07, 0.11]	0.008 [-0.08, 0.1]
Imagination (Promotion-Self)	-0.015 [-0.11, 0.08]	-0.035 [-0.13, 0.05]
Imagination (Promotion-Other)	0.044 [-0.05, 0.13]	0.017 [-0.08, 0.11]
Action Planning (Individual)	0.022 [-0.07, 0.11]	0.011 [-0.08, 0.1]
Action Planning (Collective)	0.094 [0, 0.18]	0.069 [-0.02, 0.16]
Letter to Future Gen	0.177 [0.09, 0.26]	0.148 [0.06, 0.24]
Impact Information (Text)	0.002 [-0.09, 0.09]	0.007 [-0.08, 0.09]
Impact Information (Quiz)	0.012 [-0.08, 0.1]	-0.007 [-0.09, 0.08]
Carbon Footprint (General)	-0.003 [-0.09, 0.08]	-0.054 [-0.14, 0.03]
Carbon Footprint (Personalized)	-0.054 [-0.15, 0.03]	-0.09 [-0.18, 0]
Personal Benefits	0.055 [-0.04, 0.14]	0.022 [-0.06, 0.12]

Table S11. Estimated treatment effects (Intervention – Control, comparing medians of posterior distributions) for temporal, geographic, and social aspects of psychological distance pertaining to climate change.

Contrast (intervention > control)	Temporal<br>Estimate [95% CI]	Geographic<br>Estimate [95% CI]	Social<br>Estimate [95% CI]
News Comments (Self-Rel)	-0.081 [-0.2, 0.04]	0.025 [-0.09, 0.15]	-0.012 [-0.13, 0.11]
News Comments (Social-Rel)	-0.101 [-0.22, 0.02]	-0.038 [-0.15, 0.08]	-0.047 [-0.16, 0.07]
Social Norms (Text)	0.002 [-0.11, 0.12]	0.01 [-0.11, 0.13]	0.017 [-0.09, 0.14]
Social Norms (Quiz)	0.015 [-0.11, 0.13]	-0.102 [-0.22, 0.01]	-0.187 [-0.3, -0.07]
Moral Values	-0.007 [-0.12, 0.11]	0.022 [-0.09, 0.15]	-0.133 [-0.25, -0.02]
Imagination (Prevention-Self)	0.037 [-0.08, 0.16]	0.036 [-0.08, 0.16]	0.068 [-0.05, 0.19]
Imagination (Prevention-Other)	0.104 [-0.02, 0.22]	0.073 [-0.05, 0.19]	-0.066 [-0.19, 0.06]
Imagination (Promotion-Self)	-0.037 [-0.16, 0.08]	0.033 [-0.08, 0.16]	-0.097 [-0.22, 0.03]
Imagination (Promotion-Other)	-0.081 [-0.2, 0.05]	0.022 [-0.1, 0.14]	-0.064 [-0.19, 0.05]
Action Planning (Individual)	-0.025 [-0.15, 0.09]	-0.031 [-0.15, 0.1]	-0.05 [-0.17, 0.07]
Action Planning (Collective)	-0.034 [-0.16, 0.09]	-0.057 [-0.18, 0.06]	-0.046 [-0.16, 0.08]
Letter to Future Gen	-0.018 [-0.14, 0.1]	-0.016 [-0.13, 0.11]	-0.015 [-0.14, 0.1]
Impact Information (Text)	-0.105 [-0.23, 0.01]	-0.024 [-0.14, 0.09]	0.005 [-0.11, 0.12]
Impact Information (Quiz)	-0.019 [-0.14, 0.1]	0.054 [-0.06, 0.17]	-0.02 [-0.14, 0.09]
Carbon Footprint (General)	-0.043 [-0.16, 0.07]	0.036 [-0.08, 0.15]	-0.008 [-0.13, 0.11]
Carbon Footprint (Personalized)	-0.022 [-0.15, 0.09]	0.075 [-0.04, 0.19]	-0.021 [-0.14, 0.1]
Personal Benefits	-0.17 [-0.3, -0.05]	0.029 [-0.1, 0.15]	-0.004 [-0.12, 0.12]

Table S12. Estimated treatment effects (Intervention – Control, comparing medians of posterior distributions) for climate change self-efficacy scale scores.

**Contrast (intervention > control)**	**Estimate [95% CI]**
News Comments (Self-Rel)	0.048 [-0.08, 0.16]
News Comments (Social-Rel)	0.13 [0.01, 0.25]
Social Norms (Text)	0.116 [-0.01, 0.23]
Social Norms (Quiz)	0.089 [-0.03, 0.2]
Moral Values	0.187 [0.07, 0.3]
Imagination (Prevention-Self)	0.097 [-0.02, 0.22]
Imagination (Prevention-Other)	0.047 [-0.07, 0.17]
Imagination (Promotion-Self)	0.108 [-0.01, 0.23]
Imagination (Promotion-Other)	0.153 [0.04, 0.28]
Action Planning (Individual)	0.096 [-0.02, 0.21]
Action Planning (Collective)	0.133 [0.02, 0.25]
Letter to Future Gen	0.177 [0.06, 0.29]
Impact Information (Text)	0.052 [-0.06, 0.18]
Impact Information (Quiz)	0.148 [0.03, 0.26]
Carbon Footprint (General)	0.119 [0, 0.23]
Carbon Footprint (Personalized)	-0.022 [-0.14, 0.09]
Personal Benefits	0.19 [0.07, 0.31]

Table S13. Estimated treatment effects (Intervention – Control, comparing medians of posterior distributions) for scores on the Climate Change Concern and Perceived Risk scale.

**Contrast (intervention > control)**	**Estimate [95% CI]**
News Comments (Self-Rel)	0.066 [-0.06, 0.18]
News Comments (Social-Rel)	0.094 [-0.02, 0.22]
Social Norms (Text)	-0.033 [-0.15, 0.08]
Social Norms (Quiz)	-0.024 [-0.14, 0.09]
Moral Values	0.039 [-0.08, 0.15]
Imagination (Prevention-Self)	0.188 [0.06, 0.3]
Imagination (Prevention-Other)	0.123 [0, 0.24]
Imagination (Promotion-Self)	-0.008 [-0.13, 0.12]
Imagination (Promotion-Other)	0.081 [-0.03, 0.21]
Action Planning (Individual)	0.11 [-0.01, 0.23]
Action Planning (Collective)	0.105 [-0.01, 0.22]
Letter to Future Gen	0.108 [-0.01, 0.22]
Impact Information (Text)	0.043 [-0.08, 0.16]
Impact Information (Quiz)	-0.014 [-0.13, 0.1]
Carbon Footprint (General)	0.027 [-0.09, 0.14]
Carbon Footprint (Personalized)	-0.066 [-0.19, 0.05]
Personal Benefits	0.072 [-0.05, 0.19]

Table S14. Estimated treatment effects (Intervention – Control, comparing medians of posterior distributions) for emotions related to climate change (anger, anxiety, sadness, hope, hopelessness, uncertainty, and disengagement).

Contrast (intervention > control)	**Anger** Estimate [95% CI]	**Anxiety** Estimate [95% CI]	**Sadness** Estimate [95% CI]	**Determination** Estimate [95% CI]
1. News Comments (Self-Rel)	0.029 [-0.09, 0.16]	-0.095 [-0.21, 0.02]	-0.062 [-0.18, 0.05]	0.076 [-0.04, 0.2]
2. News Comments (Social-Rel)	0.024 [-0.1, 0.15]	-0.024 [-0.14, 0.1]	0.024 [-0.09, 0.15]	0.139 [0.02, 0.25]
3. Social Norms (Text)	-0.01 [-0.13, 0.11]	-0.035 [-0.14, 0.09]	-0.047 [-0.16, 0.07]	0.062 [-0.05, 0.18]
4. Social Norms (Quiz)	-0.068 [-0.19, 0.05]	-0.105 [-0.22, 0.01]	-0.114 [-0.23, 0]	0.069 [-0.05, 0.18]
5. Moral Values	-0.025 [-0.14, 0.1]	-0.123 [-0.24, -0.01]	-0.038 [-0.15, 0.08]	0.15 [0.04, 0.27]
6. Imagination (Prevention-Self)	0.231 [0.11, 0.35]	0.126 [0.01, 0.25]	0.189 [0.07, 0.31]	0.126 [0, 0.24]
7. Imagination (Prevention-Other)	0.16 [0.04, 0.28]	0.081 [-0.03, 0.2]	0.08 [-0.03, 0.21]	0.032 [-0.09, 0.15]
8. Imagination (Promotion-Self)	-0.002 [-0.12, 0.12]	-0.033 [-0.16, 0.08]	-0.057 [-0.18, 0.06]	0.144 [0.03, 0.27]
9. Imagination (Promotion-Other)	0.036 [-0.09, 0.15]	0.01 [-0.1, 0.13]	0.025 [-0.1, 0.14]	0.131 [0.02, 0.25]
10. Action Planning (Individual)	-0.002 [-0.12, 0.12]	0.011 [-0.11, 0.13]	0.047 [-0.08, 0.16]	0.177 [0.06, 0.29]
11. Action Planning (Collective)	0.021 [-0.1, 0.14]	0.011 [-0.11, 0.14]	0.025 [-0.1, 0.14]	0.124 [0.01, 0.24]
12. Letter to Future Gen	0.129 [0.01, 0.25]	0.074 [-0.04, 0.19]	0.027 [-0.1, 0.14]	0.23 [0.11, 0.35]
13. Impact Information (Text)	-0.026 [-0.15, 0.09]	-0.057 [-0.17, 0.06]	-0.062 [-0.18, 0.05]	0.063 [-0.05, 0.18]
14. Impact Information (Quiz)	0.004 [-0.11, 0.12]	-0.009 [-0.12, 0.11]	-0.056 [-0.17, 0.06]	0.05 [-0.06, 0.17]
15. Carbon Footprint (General)	-0.014 [-0.13, 0.1]	0.014 [-0.1, 0.13]	0.031 [-0.09, 0.14]	0.04 [-0.07, 0.15]
16. Carbon Footprint (Personalized)	-0.071 [-0.19, 0.05]	-0.154 [-0.27, -0.04]	-0.051 [-0.17, 0.06]	0.034 [-0.08, 0.16]
17. Personal Benefits	-0.016 [-0.14, 0.11]	-0.02 [-0.14, 0.11]	0 [-0.13, 0.12]	0.07 [-0.05, 0.19]

Contrast #	**Hope** Estimate [95% CI]	**Hopelessness** Estimate [95% CI]	**Uncertainty** Estimate [95% CI]	**Disengagement** Estimate [95% CI]
1.	0.085 [-0.04, 0.21]	-0.023 [-0.14, 0.1]	-0.072 [-0.19, 0.04]	-0.052 [-0.17, 0.07]
2.	0.05 [-0.08, 0.17]	0.013 [-0.11, 0.13]	-0.041 [-0.16, 0.08]	-0.093 [-0.21, 0.03]
3.	0.045 [-0.07, 0.16]	-0.056 [-0.17, 0.06]	0.009 [-0.11, 0.12]	-0.038 [-0.15, 0.08]
4.	0.044 [-0.07, 0.16]	-0.08 [-0.2, 0.03]	-0.028 [-0.15, 0.08]	0.008 [-0.11, 0.13]

5.	0.122 [0.01, 0.24]	-0.066 [-0.18, 0.05]	-0.006 [-0.12, 0.11]	-0.114 [-0.23, 0]
6.	-0.034 [-0.15, 0.09]	0.088 [-0.03, 0.21]	0.075 [-0.04, 0.2]	-0.07 [-0.19, 0.04]
7.	-0.055 [-0.17, 0.07]	0.066 [-0.05, 0.19]	0.023 [-0.09, 0.14]	-0.013 [-0.14, 0.11]
8.	0.136 [0.02, 0.26]	-0.137 [-0.25, -0.01]	-0.03 [-0.15, 0.09]	-0.037 [-0.15, 0.09]
9.	0.062 [-0.06, 0.19]	-0.062 [-0.18, 0.06]	0.074 [-0.05, 0.19]	-0.06 [-0.18, 0.06]
10.	0.074 [-0.04, 0.2]	-0.079 [-0.19, 0.04]	-0.054 [-0.17, 0.06]	-0.082 [-0.2, 0.04]
11.	0.092 [-0.03, 0.21]	0.005 [-0.12, 0.12]	-0.015 [-0.14, 0.1]	-0.113 [-0.23, 0.01]
12.	0.191 [0.07, 0.31]	-0.002 [-0.12, 0.12]	0.02 [-0.1, 0.14]	-0.141 [-0.26, -0.02]
13.	0.014 [-0.1, 0.13]	-0.059 [-0.17, 0.06]	0.011 [-0.11, 0.12]	-0.042 [-0.16, 0.07]
14.	0.026 [-0.1, 0.14]	-0.025 [-0.14, 0.1]	-0.028 [-0.14, 0.09]	-0.074 [-0.19, 0.04]
15.	-0.044 [-0.16, 0.07]	0.023 [-0.09, 0.14]	0.049 [-0.07, 0.16]	-0.063 [-0.19, 0.05]
16.	0.04 [-0.08, 0.16]	-0.085 [-0.2, 0.03]	-0.155 [-0.28, -0.04]	-0.092 [-0.21, 0.03]
17.	0.099 [-0.02, 0.23]	-0.015 [-0.13, 0.11]	-0.057 [-0.18, 0.06]	-0.043 [-0.16, 0.08]

Table S15. Estimated treatment effects (Intervention – Control, comparing medians of posterior distributions) for signing-related outcome measures obtained from the Petitions Task (signing intention, clicks on petition links, and post-hoc self-reported signing).

**Contrast (intervention > control)**	**Signing Intention**<br>Estimate [95% CI]	**Clicks**<br>Estimate [95% CI]	**Self-Reported Signing**<br>Estimate [95% CI]
News Comments (Self-Rel)	0.051 [-0.05, 0.16]	0.068 [-0.05, 0.19]	0.111 [-0.49, 0.75]
News Comments (Social-Rel)	0.106 [0, 0.21]	0.075 [-0.05, 0.19]	0.238 [-0.38, 0.83]
Social Norms (Text)	0.001 [-0.1, 0.11]	-0.072 [-0.19, 0.04]	-0.188 [-0.82, 0.39]
Social Norms (Quiz)	-0.042 [-0.15, 0.06]	0.003 [-0.11, 0.11]	-0.398 [-1, 0.25]
Moral Values	0.063 [-0.04, 0.16]	0.227 [0.12, 0.35]	0 [-0.61, 0.58]
Imagination (Prevention-Self)	0.029 [-0.07, 0.14]	-0.011 [-0.14, 0.11]	-0.52 [-1.16, 0.12]
Imagination (Prevention-Other)	-0.009 [-0.11, 0.1]	0.002 [-0.12, 0.12]	-0.03 [-0.68, 0.57]
Imagination (Promotion-Self)	-0.068 [-0.17, 0.04]	0.004 [-0.12, 0.12]	-0.189 [-0.85, 0.43]
Imagination (Promotion-Other)	0.011 [-0.09, 0.12]	-0.054 [-0.17, 0.07]	-0.224 [-0.85, 0.42]
Action Planning (Individual)	-0.004 [-0.12, 0.1]	-0.023 [-0.14, 0.1]	-0.004 [-0.64, 0.63]
Action Planning (Collective)	0.092 [-0.01, 0.2]	0.013 [-0.11, 0.13]	-0.132 [-0.74, 0.51]
Letter to Future Gen	0.216 [0.12, 0.33]	0.231 [0.11, 0.35]	-0.136 [-0.78, 0.48]
Impact Information (Text)	0.003 [-0.1, 0.11]	0.047 [-0.07, 0.16]	0.09 [-0.5, 0.72]
Impact Information (Quiz)	0.065 [-0.04, 0.17]	0.012 [-0.11, 0.13]	-0.16 [-0.77, 0.42]
Carbon Footprint (General)	-0.066 [-0.17, 0.04]	-0.051 [-0.17, 0.07]	-0.282 [-0.9, 0.34]
Carbon Footprint (Personalized)	-0.074 [-0.18, 0.03]	-0.099 [-0.22, 0.02]	-0.143 [-0.77, 0.45]
Personal Benefits	0.152 [0.05, 0.26]	0.166 [0.04, 0.28]	-0.246 [-0.88, 0.37]

Table S16. Estimated treatment effects (Intervention – Control, comparing medians of posterior distributions) for intentions to engage in pro-environmental behaviors. This model includes all trials, even "maxed-out" trials where participants reported already engaging in a target behavior at the highest possible frequency (e.g., always eating vegetarian meals; never driving a car). These estimates can be compared with Table S2, which reports similar results with "maxed-out" trials excluded.

**Contrast (intervention > control)**	**Estimate [95% CI]**
News Comments (Self-Rel)	0.024 [-0.04, 0.08]
News Comments (Social-Rel)	0.054 [-0.01, 0.12]
Social Norms (Text)	0.005 [-0.06, 0.06]
Social Norms (Quiz)	0.025 [-0.04, 0.08]
Moral Values	0.077 [0.02, 0.14]
Imagination (Prevention-Self)	0.14 [0.08, 0.21]
Imagination (Prevention-Other)	0.078 [0.02, 0.14]
Imagination (Promotion-Self)	0.046 [-0.02, 0.11]
Imagination (Promotion-Other)	0.05 [-0.01, 0.11]
Action Planning (Individual)	0.103 [0.04, 0.16]
Action Planning (Collective)	0.066 [0, 0.13]
Letter to Future Gen	0.135 [0.07, 0.2]
Impact Information (Text)	0.03 [-0.03, 0.09]
Impact Information (Quiz)	0.045 [-0.02, 0.1]
Carbon Footprint (General)	0.017 [-0.04, 0.08]
Carbon Footprint (Personalized)	-0.01 [-0.07, 0.05]
Personal Benefits	0.099 [0.04, 0.16]

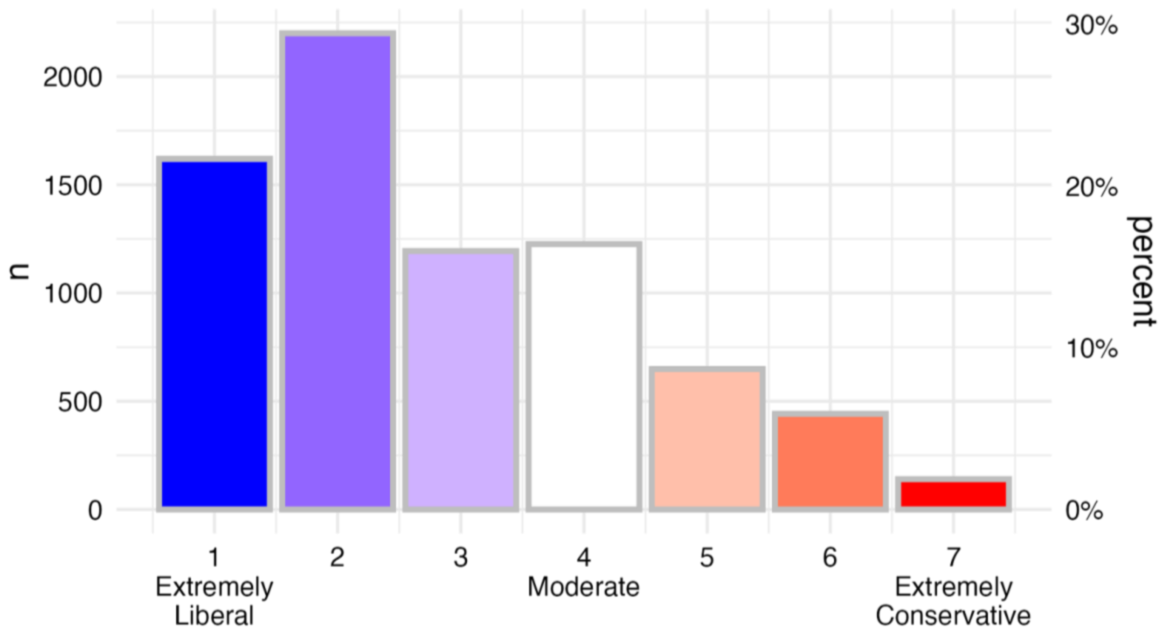

Figure S1. Distribution of self-reported political ideology among participants in the final sample, after exclusions.

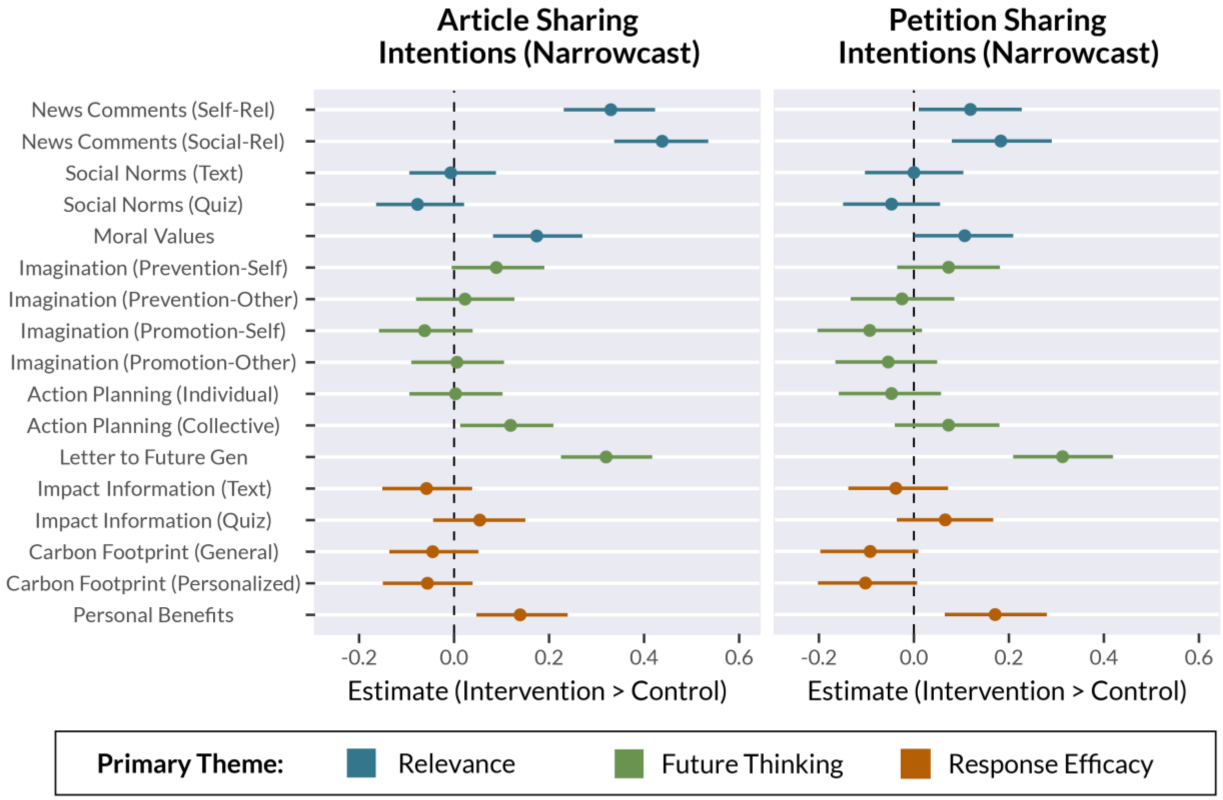

Figure S2. Intentions to share news articles and petitions about climate change directly with known others ("narrowcast" sharing). Results are similar to broadcast sharing results, reported in the main text (Figure 4). Results shown are estimates derived from Bayesian mixed-effects regression models. Point estimates indicate the treatment effect for each intervention condition (Intervention–Control, comparing the median values from each posterior distribution). Error bars mark 95% credible intervals surrounding the point estimates. Dependent variables were z-scored to provide standardized effect sizes. Dotted lines marks zero (no effect; no difference from Control group). Points are color-coded to reflect the three intervention themes: Relevance, Future Thinking, and Response Efficacy. Note that some interventions can be described by more than one theme; colors here indicate the primary theme for each intervention.

SI References

1. S. Permut, M. Fisher, D. M. Oppenheimer, TaskMaster: A Tool for Determining When Subjects Are on Task. *Adv. Methods Pract. Psychol. Sci.* **2**, 188–196 (2019).

2. L. S. Loy, F. Wieber, P. M. Gollwitzer, G. Oettingen, Supporting Sustainable Food Consumption: Mental Contrasting with Implementation Intentions (MCII) Aligns Intentions and Behavior. *Front. Psychol.* **7** (2016).

3. J. A. Martenstyn, A. M. Grant, An online, comparative effectiveness trial of mental contrasting with implementation intentions (MCII) versus solution-focused coaching (SFC) questions. *Coach. Int. J. Theory Res. Pract.* **15**, 60–84 (2022).

4. A. Ort, A. Fahr, Mental contrasting with implementation intentions as a technique for media-mediated persuasive health communication. *Health Psychol. Rev.* **16**, 602–621 (2022).

5. G. Oettingen, P. M. Gollwitzer, Strategies of setting and implementing goals : Mental contrasting and implementation intentions. (2010).

6. M. Vlasceanu, *et al.*, Addressing climate change with behavioral science: A global intervention tournament in 63 countries. *Sci. Adv.* **10**, eadj5778 (2024).

7. D. Cosme, *et al.*, Message self and social relevance increases intentions to share content: Correlational and causal evidence from six studies. *J. Exp. Psychol. Gen.* **152**, 253–267 (2023).

8. A. Tyson, C. Funk, B. Kennedy, What the data says about Americans' views of climate change. *Pew Res. Cent.* (2023). Available at: https://www.pewresearch.org/short-reads/2023/08/09/what-the-data-says-about-americans-views-of-climate-change/ [Accessed 2 September 2024].

9. A. T. and B. Kennedy, How Americans View Future Harms From Climate Change in Their Community and Around the U.S. *Pew Res. Cent.* (2023). Available at: https://www.pewresearch.org/science/2023/10/25/how-americans-view-future-harms-from-climate-change-in-their-community-and-around-the-u-s/ [Accessed 2 September 2024].

10. A. Shaw, Poll Points to Growing Awareness of Sustainability. *Kitchen Bath Des. News* (2023). Available at: https://www.kitchenbathdesign.com/survey-points-to-increased-awareness-of-sustainability/ [Accessed 2 September 2024].

11. Ipsos, "Americans are Sick of Single-Use Plastic Pollution, Poll Finds" (Oceana, 2023).

12. Z. Harris, A Large Majority of Americans Are Willing to Pay More for Sustainable Products, Study Finds. *BusinessWire* (2022). Available at: https://www.businesswire.com/news/home/20220621005065/en/A-Large-Majority-of-Americans-Are-Willing-to-Pay-More-for-Sustainable-Products-Study-Finds [Accessed 2 September 2024].

13. K. Lacroix, *et al.*, PLANTS: A scalable survey tool for identifying groups willing to adopt plant-based diets. [Preprint] (2022). Available at: https://osf.io/hkcvx [Accessed 2 September 2024].

14. M. Atari, *et al.*, Morality beyond the WEIRD: How the nomological network of morality varies across cultures. *J. Pers. Soc. Psychol.* **125**, 1157–1188 (2023).

15. D. Ivanova, *et al.*, Quantifying the potential for climate change mitigation of consumption options. *Environ. Res. Lett.* **15**, 093001 (2020).

16. US Department of Energy, Fuel Economy Web Services.

17. N. Kommenda, How your flight emits as much CO2 as many people do in a year. *The Guardian* (2019).

18. U.S. Environmental Protection Agency, Carbon Footprint Calculator. *US Environ. Prot. Agency* (2016). Available at: https://www3.epa.gov/carbon-footprint-calculator [Accessed 2 September 2024].

19. U.S. Environmental Protection Agency, eGRID with 2022 Data. Deposited 17 May 2022.

20. H. Bastawrose, T. Warren, Average Energy Consumption per Household [2024 U.S Study] - EnergyBot. *EnergyBot* (2024). Available at: https://www.energybot.com/blog/average-energy-consumption.html [Accessed 2 September 2024].

21. U.S. Environmental Protection Agency, Greenhouse Gas Emissions from a Typical Passenger Vehicle. *US Environ. Prot. Agency* (2016). Available at: https://www.epa.gov/greenvehicles/greenhouse-gas-emissions-typical-passenger-vehicle [Accessed 2 September 2024].

22. H. Ritchie, Where in the world do people have the highest CO2 emissions from flying? *Our World Data* (2020).

23. D. Ivanova, *et al.*, Quantifying the potential for climate change mitigation of consumption options. *Environ. Res. Lett.* **15**, 093001 (2020).

24. R. Christensen, G. Knezek, The Climate Change Attitude Survey: Measuring Middle School Student Beliefs and Intentions to Enact Positive Environmental Change. *Int. J. Environ. Sci. Educ.* **10**, 773–788 (2015).

25. A. Spence, W. Poortinga, N. Pidgeon, The Psychological Distance of Climate Change. *Risk Anal.* **32**, 957–972 (2012).

26. N. Weinstein, A. K. Przybylski, R. M. Ryan, The index of autonomous functioning: Development of a scale of human autonomy. *J. Res. Personal.* **46**, 397–413 (2012).

27. S. Clayton, B. T. Karazsia, Development and validation of a measure of climate change anxiety. *J. Environ. Psychol.* **69**, 101434 (2020).

28. P.-C. Bürkner, brms : An R Package for Bayesian Multilevel Models Using Stan. *J. Stat. Softw.* **80** (2017).

29. R. V. Lenth, emmeans: Estimated Marginal Means, aka Least-Squares Means. (2021). Deposited 2021.

30. H. Wickham, *ggplot2: Elegant Graphics for Data Analysis* (Springer-Verlag New York, 2016).

31. H. Wickham, R. François, L. Henry, K. Müller, D. Vaughan, *dplyr: A Grammar of Data Manipulation* (2023).

32. H. Wickham, D. Vaughan, M. Girlich, *tidyr: Tidy Messy Data* (2024).

33. H. Wickham, *stringr: Simple, Consistent Wrappers for Common String Operations* (2023).

34. D. J. Barr, R. Levy, C. Scheepers, H. J. Tily, Random effects structure for confirmatory hypothesis testing: Keep it maximal. *J. Mem. Lang.* **68**, 10.1016/j.jml.2012.11.001 (2013).

35. H. Matuschek, R. Kliegl, S. Vasishth, H. Baayen, D. Bates, Balancing Type I error and power in linear mixed models. *J. Mem. Lang.* **94**, 305–315 (2017).
